# Structures of mammalian GLD-2 proteins reveal molecular basis of their functional diversity in mRNA and microRNA processing

**DOI:** 10.1093/nar/gkaa578

**Published:** 2020-07-07

**Authors:** Xiao-Yan Ma, Hong Zhang, Jian-Xiong Feng, Jia-Li Hu, Bing Yu, Li Luo, Yu-Lu Cao, Shuang Liao, Jichang Wang, Song Gao

**Affiliations:** State Key Laboratory of Oncology in South China, Collaborative Innovation Center for Cancer Medicine, Sun Yat-sen University Cancer Center, Guangzhou, Guangdong 510060, China; State Key Laboratory of Oncology in South China, Collaborative Innovation Center for Cancer Medicine, Sun Yat-sen University Cancer Center, Guangzhou, Guangdong 510060, China; State Key Laboratory of Oncology in South China, Collaborative Innovation Center for Cancer Medicine, Sun Yat-sen University Cancer Center, Guangzhou, Guangdong 510060, China; State Key Laboratory of Oncology in South China, Collaborative Innovation Center for Cancer Medicine, Sun Yat-sen University Cancer Center, Guangzhou, Guangdong 510060, China; State Key Laboratory of Oncology in South China, Collaborative Innovation Center for Cancer Medicine, Sun Yat-sen University Cancer Center, Guangzhou, Guangdong 510060, China; State Key Laboratory of Oncology in South China, Collaborative Innovation Center for Cancer Medicine, Sun Yat-sen University Cancer Center, Guangzhou, Guangdong 510060, China; State Key Laboratory of Oncology in South China, Collaborative Innovation Center for Cancer Medicine, Sun Yat-sen University Cancer Center, Guangzhou, Guangdong 510060, China; State Key Laboratory of Oncology in South China, Collaborative Innovation Center for Cancer Medicine, Sun Yat-sen University Cancer Center, Guangzhou, Guangdong 510060, China; Key Laboratory for Stem Cells and Tissue Engineering (Sun Yat-sen University), Ministry of Education, Guangzhou 510080, China; Department of histology and embryology, Zhongshan School of Medicine, Sun Yat-sen University, Guangzhou 510080, China; State Key Laboratory of Oncology in South China, Collaborative Innovation Center for Cancer Medicine, Sun Yat-sen University Cancer Center, Guangzhou, Guangdong 510060, China; Guangzhou Regenerative Medicine and Health Guangdong Laboratory, Guangzhou 510530, China

## Abstract

The stability and processing of cellular RNA transcripts are efficiently controlled via non-templated addition of single or multiple nucleotides, which is catalyzed by various nucleotidyltransferases including poly(A) polymerases (PAPs). Germline development defective 2 (GLD-2) is among the first reported cytoplasmic non-canonical PAPs that promotes the translation of germline-specific mRNAs by extending their short poly(A) tails in metazoan, such as *Caenorhabditis elegans* and *Xenopus*. On the other hand, the function of mammalian GLD-2 seems more diverse, which includes monoadenylation of certain microRNAs. To understand the structural basis that underlies the difference between mammalian and non-mammalian GLD-2 proteins, we determine crystal structures of two rodent GLD-2s. Different from *C*. *elegans* GLD-2, mammalian GLD-2 is an intrinsically robust PAP with an extensively positively charged surface. Rodent and *C*. *elegans* GLD-2s have a topological difference in the β-sheet region of the central domain. Whereas *C*. *elegans* GLD-2 prefers adenosine-rich RNA substrates, mammalian GLD-2 can work on RNA oligos with various sequences. Coincident with its activity on microRNAs, mammalian GLD-2 structurally resembles the mRNA and miRNA processor terminal uridylyltransferase 7 (TUT7). Our study reveals how GLD-2 structurally evolves to a more versatile nucleotidyltransferase, and provides important clues in understanding its biological function in mammals.

## INTRODUCTION

Poly(A) polymerases (PAPs) catalyze non-templated addition of adenosines to the 3′ terminus of mRNAs (1,2). Eukaryotic PAPs can be classified into two subgroups, namely canonical PAPs and non-canonical PAPs. Canonical PAPs, represented by PAPα, are mainly responsible for the polyadenylation of pre-mRNAs in the nucleus (3). Non-canonical PAPs have been found to polyadenylate specific mRNAs in nucleus and/or cytoplasm (4–7). As the first discovered non-canonical PAP in metazoan, germline development defective 2 (GLD-2, or PAP-associated domain-containing protein 4 [PAPD4], or terminal nucleotidyltransferase 2 [TENT2] in vertebrates) was initially reported in *Caenorhabditis elegans* and subsequently also found in other species (6,8–11), with functional implication in re-extending short poly(A) tails in the cytoplasm of certain cells (6). Cytoplasmic polyadenylation by GLD-2 counteracts deadenylation of poly(A) tails that usually causes mRNA decay, thereby enhancing the stability of mRNAs (12,13). In *C. elegans, Drosophila* and *Xenopus*, this GLD-2-mediated translational regulation mechanism is suggested to ensure the efficiency of protein synthesis in oocytes and early embryos, where transcription is silenced (8,14–16).

GLD-2 proteins from different species have various lengths, but share a conserved nucleotidyltransferase (NTase) region composed of a catalytic domain and a central domain. The closest GLD-2 relative in yeast is the cytoplasmic NTase Caffeine-induced death protein 1 (Cid1) that catalyzes polyuridylation of mRNA (17). The PAP activity of *C. elegans* GLD-2 alone is extremely weak, but can be stimulated by other protein partners such as GLD-3 and RRM domain-containing protein 8 (RNP-8) (6,18–19). Structural studies reveal that GLD-3 or RNP-8 wraps around the backside of the catalytic center of GLD-2, so as to stabilize the overall architecture and provide a positively charged area required for substrate RNA binding (20,21). According to WormBase (22), no mammalian homolog of GLD-3 is found. And RNP-8 has two orthologs in mammals, namely RNA-binding protein 7 (RBM7) and RBM11. However, these two proteins share very low sequences similarity to RNP-8, and no evidence shows they can interact with GLD-2. In *Xenopus*, GLD-2 forms cytoplasmic polyadenylation machinery with cleavage and polyadenylation specificity factor (CPSF), cytoplasmic polyadenylation element binding protein (CPEB), symplekin and other proteins (15). These partners, existing also in mammals, are suggested to facilitate the recognition of specific motifs in mRNA, but not to enhance their enzymatic activity (23). Thus, whether mammalian GLD-2 requires GLD-3/RNP-8-like partner for the stimulation of PAP activity is unclear.

Functions of mammalian GLD-2s seem quite different from their homologs in non-mammals. While GLD-2 is essential for gametogenesis in *C. elegans* and *Drosophila* (9,14), GLD-2-deficient mice have normal fertility (23). Another feature of mammalian GLD-2 is the ability to modulate 3′ terminal extension of microRNAs (miRNAs). GLD-2 is responsible for adding single adenosine at the 3′-end of miRNAs, such as miRNA-122 (24–27). In THP-1, HCT-116 and other human cell lines, knockdown of GLD-2 was shown to correlate with subdued 3′ adenylation and hence the decreased transcription, of specific miRNAs (28,29). Moreover, GLD-2 was found to potentially monouridylate pre-miRNAs during the biogenesis of group II let-7 miRNAs to promote their stability (30). It is also suggested that GLD-2 oligouridylates pre-miRNAs with a 5′ overhang, which leads to the degradation of these abnormal miRNAs (28,30,31). While *C. elegans* GLD-2 was reported to preferentially work on adenosine-rich RNAs (20), how mammalian GLD-2 acquires the ability to mediate miRNA processing during evolution remains unknown.

In this study, we have purified the NTase regions of several mammalian GLD-2s, and find that they exhibit substantially higher *in vitro* PAP activity as compared to *C. elegans* GLD-2. Structural analysis reveals an extensively positively charged surface of mammalian GLD-2 that facilitates the binding of substrate RNA. Unlike *C. elegans* GLD-2 which specifically adenylates poly(A) primers, mammalian GLD-2 works on RNA substrates with various sequences. Further structural comparison implicates a prominent similarity between mammalian GLD-2 and human TUT7. Finally, we tested positively charged surface residues of mammalian GLD-2 that may be involved in binding of RNA substrates. These findings reveal special evolutionary features of mammalian GLD-2 and advance the understanding of their cellular functions.

## MATERIALS AND METHODS

### Construct information

cDNAs of *Homo sapiens* (hs), *Mus musculus* (mm) and *Rattus norvegicus* (rn)GLD-2 (NCBI accession numbers NM_00114394, NM_001361537 and NM_001008372, respectively) were purchased from YouBio Biological Company (China). The NTase regions of mammalian GLD-2s were individually cloned into a modified pET28 vector with an N-terminal His_6_-tag followed by a PreScission protease (PSP) cleavage site. Recombinant pET-22b-ceGLD-2 encoding the core PAP region of *C*. *elegans* (ce)GLD-2 (NM_059441), containing residues 526−923 with an internal deletion of 813−847, were purchased from General Biosystems (China). Full-length hsGLD-2 was cloned into the pCAGGS vector with an N-terminal Flag-tag. Full-length hsGLD-2(D279A) was cloned into a modified pET30a vector with an N-terminal His_6_-tag and an MBP-tag followed by a PSP cleavage site, respectively. All constructs were validated by sequencing.

### Protein expression and purification

The NTase region of mammalian GLD-2 was expressed in *Escherichia coli Rosetta* (DE3) cells (Invitrogen). Transformed bacteria were cultured at 37°C in Terrific Broth (TB) medium before induced with 0.5 mM isopropyl-1-thio-β-D-galactopyranoside (IPTG) at an OD_600 nm_ of 0.6, and grown overnight at 18°C. The cells were lysed in 20 mM Tris–HCl, pH 8.0, 300 mM NaCl, 10 mM imidazole, 1 mM phenylmethanesulfonyl fluoride (PMSF), 1 μM DNase I and 2 mM β-Mercaptoethanol (β-ME) using a cell disruptor (JNBIO) and subjected to centrifugation at 40 000 *g* for 1 h. The supernatant was filtered and applied to a Ni-NTA column (GE Healthcare). The Ni-NTA purification was carried out in 20 mM Tris–HCl, pH 8.0, 300 mM NaCl and 2 mM β-ME (buffer A), and 10, 30 and 300 mM imidazole was added for equilibration, wash and elution, respectively. For rnGLD-2_131-484_(D279A) and mmGLD-2_143-484_(D213A/D279A) that yielded crystals, proteins were subsequently loaded onto a HiLoad 16/600 Superdex 200 pg column (GE Healthcare) equilibrated with 20 mM Tris–HCl, pH 8.0, 1.5 M NaCl and 2 mM dithiothreitol (DTT). Peak fractions of the target protein were collected, concentrated and applied to a second size exclusion chromatography using the same column in 20 mM Tris–HCl, pH 8.0, 150 mM NaCl and 2 mM DTT. Other recombinant proteins were incubated with 20 μg glutathione-S-transferase (GST)-fused PSP to remove the His_6_-tag and dialyzed overnight against buffer A. After dialysis, PSP was removed using a GST column. The protein was re-applied to a second Ni-NTA column equilibrated with buffer A, and eluted with extra 50 mM imidazole. Proteins were subsequently loaded onto a HiLoad 16/600 Superdex 200 pg column (GE Healthcare) equilibrated with 20 mM Tris–HCl, pH 8.0, 300 mM NaCl, 2 mM MgCl_2_ and 2 mM DTT. Full-length hsGLD-2(D279A) were expressed and purified in the same way as the NTase region stated above with pH 7.4 buffers. The tandem His_6_-tag and MBP-tag were removed by PSP cleavage. For the expression of full-length hsGLD-2, 293T cell cultured in 10-cm dishes were transfected with 20 µg pCAGGS-Flag-hsGLD-2 using Lipofectamine 2000 (Invitrogen). Forty eight hours after transfection, cells were harvested and lysed on ice for 30 min in a buffer B (20 mM Tris–HCl pH 7.4, 150 mM NaCl, 2 mM MgCl_2_) plus 1 × Protease Inhibitor Cocktail (TagetMol), and 1% Triton X-100 (Anatrace). After centrifugation at 23 000 *g* for 20 min, the supernatant was mixed with 100 µl of Anti-DYKDDDDK G1 Affinity Resin (GenScript) and rotated for 2 h at 4°C. The beads were washed three times with buffer B, and eluted with an extra 300 µg/ml DYKDDDDK peptide in buffer B.

### Protein crystallization

Crystallization was carried out at 18°C via hanging drop vapor diffusion by mixing equal volumes of protein (approximately 15 mg ml^−1^) and reservoir solution. Crystals of rnGLD-2_131-484_(D279A) were obtained from 0.1 M Tris–HCl, pH 8.0 and 22.4% PEG3350. Crystals of mmGLD-2_143-484_(D213A/D279A) grew from 0.08 M HEPES, pH 7.4, 23.6% PEG3350. Crystals were flash-cooled in liquid nitrogen for X-ray diffraction analysis.

### Data collection and structure determination

All diffraction datasets were collected at beamline BL17U1 and BL19U1 of the Shanghai Synchrotron Radiation Facility (SSRF) (32) and processed with the XDS suite (33). Phases were obtained by molecular replacement with MRBUMP (34) from the CCP4 package (35), and the ceGLD-2 structure (PDB code 5jnb) was applied as a search model. Models were built with COOT (36) and refined with PHENIX (37). Structural validation was carried out using MolProbity (38). The Ramachandran statistics are: 96.4% in favoured region, 3.6% allowed, 0 outlier for rnGLD-2_131-484_(D279A); 98.0% favored, 2.0% allowed, 0 outlier for mmGLD-2_143-484_(D213A/D279A). Structural illustrations were prepared using the PyMOL Molecular Graphics System (version 2.0.4, Schrödinger LLC; http://www.pymol.org/). Key parameters and statistics for X-ray diffraction data collection and refinement were summarized in Table 1.

**Table 1.** Data collection and refinement statistics

	mmGLD-2	rnGLD-2
**Data collection**		
Space group	P 1	P 12_1_1
Cell dimensions		
*a*, *b*, *c* (Å)	42.28, 47.14, 105.15	79.96, 40.72, 105.90
α, β, γ (°)	87.38, 88.06, 63.81	90, 100.20, 90
Wavelength (Å)	0.91800	0.97776
Resolution (Å)*	42.28–2.70 (2.80–2.70)	47.20–2.50 (2.58–2.50)
*R*_merge_*	0.091 (0.535)	0.050 (0.524)
CC_1/2_ (%)*	0.977 (0.835)	0.999 (0.914)
*I*/σ (*I*)*	11.2 (1.8)	21.5 (3.2)
Completeness (%)*	80.3 (78.7)	99.11 (98.84)
Redundancy*	2.6 (2.6)	6.5 (6.7)
**Refinement**		
Resolution (Å)	35.51–2.70 (2.84–2.70)	47.20–2.50 (2.55–2.50)
Unique reflections*	16 025 (1597)	23 497 (2302)
*R*_work_/*R*_free_(%)	25.3/27.1 (38.9/41.3)	20.5/24.3 (29.7/37.7)
No. atoms		
Protein	5292	5235
Water	30	39
B-factors (Å^2^)		
Protein	91.53	82.68
Water	36.93	64.50
R.m.s. deviations		
Bond lengths (Å)	0.008	0.010
Bond angles (°)	1.162	1.213
Ramachandran statistics		
Favored (%)	97.98	96.38
Outliers (%)	0	0

*Values in parentheses are for the highest-resolution shell.

### 3′-end extension assay for model RNA substrates

Unless specified, 3′ end extension assays were carried out by incubating 400 nM protein with 500 nM 5′ biotinylated 15-mer model RNA substrate and 500 μM nucleotide in a buffer containing 20 mM Tris-HCl, pH 8.0, 300 mM NaCl, 2 mM MgCl_2_ and 2 mM DTT at 37°C for 20 min. The reaction was terminated by adding 2 × RNA loading dye (NEB) and 8 μl mixture was subsequently loaded onto a 15% polyacrylamide-7M urea gel. Electrophoresis experiments were done under 180V for 90 min at room temperature. The RNAs were subsequently transferred to Hybond N^+^ membrane (GE Healthcare) under 60V for 14 min at 4°C before visualized using the Chemiluminescent Nucleic Acid Detection Module Kit (Thermo Scientific).

### Nucleotide incorporation assay

For A_15_ substrate, 400 nM protein was incubated with 500 nM RNA, 300 μM adenosinetriphosphate (ATP) and 12 μCi/mmol [α-^32^P]-ATP (PerkinElmer) in a buffer containing 20 mM Tris–HCl pH 8.0, 300 mM NaCl and 2 mM DTT, supplied with 2 mM different additives, including EDTA, MgCl_2_, MnCl_2_, ZnCl_2_, CaCl_2_, NiCl_2_ and FeSO_4_. For pre-let-7a substrate, 25, 50, 100 and 200 nM protein were individually incubated with 500 nM RNA, 300 μM UTP and 12 μCi/mmol [α-^32^P]-UTP (PerkinElmer) in a buffer containing 20 mM Tris–HCl pH 8.0, 300 mM NaCl, 2 mM MgCl_2_ and 2 mM DTT. The reaction mixture was incubated at 37°C for 20 min and terminated by adding 2 × RNA loading dye (NEB). A total of 8 μl sample was loaded onto a 20% (for A_15_) or 10% (for pre-let-7a) polyacrylamide-7M urea gel, which was exposed to a phosphorimaging plate and visualized with a Typhoon TRIO^+^ Variable Mode Imager (GE Healthcare).

### Analytical gel filtration

Analytical gel filtration experiments were carried out using a Superdex 200 10/300 column (GE Healthcare) in a buffer containing 20 mM Tris–HCl, pH 8.0, 300 mM NaCl and 2 mM MgCl_2_.

### Isothermal titration calorimetry (ITC)

Binding of rnGLD-2 to different adenosine nucleotides were performed by isothermal titration calorimetry (ITC) using a Micro CalPEAQ-ITC (Malvern) at 25°C in a buffer containing  20 mM Tris–HCl, pH 8.0 and  300 mM NaCl.  0.8 mM adenosine nucleotide was titrated at 2 μl step against 80 μM protein. Resulting heat changes upon each injection was integrated using the PEAQ-ITC program provided by the manufacturer.

### NTP consumption assay

A total of 500 nM protein was incubated with 2.5 μM RNA primer and 25 μM NTP in a buffer containing 20 mM Tris–HCl pH 8.0, 300 mM NaCl, 2 mM MgCl_2_ and 2 mM DTT at 37°C. At each time point, 10 μl sample was collected and prepared for subsequent analysis with a final volume of 25 μl. The HPLC system (Agilent) was equipped with a reverse phase C18 ODS-2 Hypersil analytical column preceded by a C18 guard column (Thermo Scientific), with 100 mM potassium phosphate pH 6.5, 10 mM tetrabutyl ammonium bromide and 10% acetonitrile as running buffer. Nucleotides were detected by absorption at 256 nm and quantified by integration of the corresponding peaks.

### miRNA 3′-end extension assay

A total of 400 nM protein was incubated with 500 nM 5′ biotinylated miRNA substrate and 500 μM nucleotide in a buffer containing 20 mM Tris–HCl, pH 8.0, 300 mM NaCl, 2 mM MgCl_2_ and 2 mM DTT at 37°C for 10 min. The following sample preparation and result analysis steps are the same as the 3′-end extension assay for model RNA substrates.

### Surface conservation plot

Protein sequences were downloaded from Uniprot (39) (https://www.uniprot.org/) and aligned using MAFFT (40) (https://www.ebi.ac.uk/Tools/msa/mafft/). Two alignment sets were performed to analyze the conservation of rnGLD-2 within the GLD-2 family and among other nucleotidyltransferases, respectively. Set 1 contains 11 GLD-2 ortholog from nine species, namely *H. sapiens* (UniProt accession Q6PIY7), *Bos taurus* (Q2HJ44), *M. musculus* (Q91YI6), *R. norvegicus* (Q5U315), *Xenopus laevis* (2 homologs, Q641A1 and Q6DFA8), *Xenopus tropicalis* (Q0VFA3), *Danio rerio* (Q503I9), *Drosophila melanogaster* (2 homologs, Q9VD44 and Q9VYS4) and *C. elegans* (O17087). Set 2 involves 10 proteins, namely 2 GLD-2 proteins from *H. sapiens* and *R. norvegicus*, TUT7 from *H. sapiens* (Q5VYS8) and *M. musculus* (Q5BLK4), TUT4 from *H. sapiens* (Q5TAX3) and *M. musculus* (B2RX14), 2 MTPAP from *H. sapiens* (Q9NVV4) and *M. musculus* (Q9D0D3), and Cid1 from *Schizosaccharomyces pombe* (O13833). The alignment results and structure of rnGLD-2 were uploaded to the online ConSurf Server (41) (http://conseq.tau.ac.il) to compute conservation scores for the residues. Surface plot of rnGLD-2 with conservation score-based coloring was generated using PyMOL Molecular Graphic Systems (version 2.0.4, Schrödinger LLC; http://www.pymol.org/).

### Electrophoretic mobility shift assay (EMSA)

A total of 4 μM protein was incubated with 50 nM 5′ biotinylated RNA primer in a buffer containing 20 mM Tris–HCl pH 8.0, and 100 mM NaCl at 37°C for 20 min. The reaction was terminated by adding 6 × native loading dye (containing 18% glycerol and 0.1% bromophenol blue). A total of 5 μl mixture was loaded onto a native polyacrylamide gel containing 6% 19:1 acrylamide/methylene bisacrylamide (Sangon Biotech), 10% 1 × TAE buffer pH 7.2, 0.1% APS and TEMED. Electrophoresis experiments were performed under 120V at 4°C. The RNAs were subsequently transferred to Hybond N^+^ membrane (GE Healthcare) under 60V for 14 min at 4°C before visualized using the Chemiluminescent Nucleic Acid Detection Module Kit (Thermo Scientific).

## RESULTS AND DISCUSSION

### Overall structure of mammalian GLD-2s

**Figure 1.**
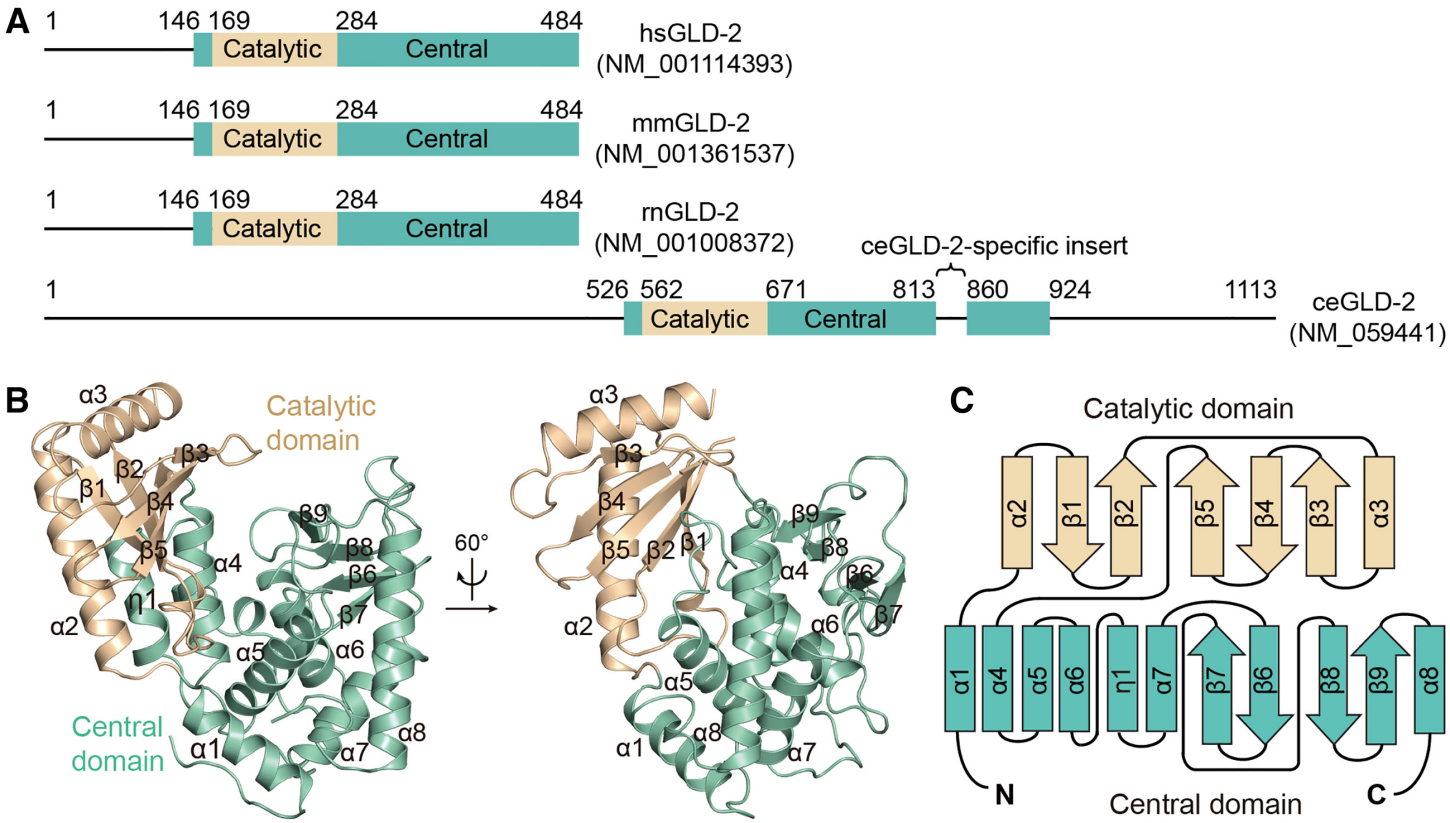
Overall structure of GLD-2. (**A**) Schematic representation showing the domain organization of mammalian and *Caenorhabditiselegans* GLD-2 homologs. Borders of the domains are indicated by residue numbers. hs, *Homo sapiens*; mm, *Mus musculus*; rn, *Rattus norvegicus*; ce, *Caenorhabditis elegans*. (**B**) Cartoon representation of rnGLD-2, colored as in A. The identity of each helix and β-strand are indicated. (**C**) The topology diagram of rnGLD-2. Secondary structural elements are not drawn to scale. Elements of rnGLD-2 are named and colored as in B.

Despite a relatively conserved NTase region, GLD-2 displays marked divergence in protein length among different species across the evolution. The NTase region of *C*. *elegans* (ce)GLD-2 is flanked by N- and C-terminal low-complexity sequences comprising around 500 and 200 amino acid residues, respectively. Compared to ceGLD-2, mammalian GLD-2s are featured by apparently shortened N- and C-termini, as well as the absence of a ceGLD-2-specific insert in the central domain (Figure 1A). We purified the NTase regions of human (hs), mouse (mm) and rat (rn)GLD-2s (residues 131–484 for all three, and for mmGLD-2 another truncated version containing residues 143–484 was used for crystallization). The NTase region of hsGLD-2 shares over 90% sequence identity to that of rodent GLD-2s, and ∼32% to ceGLD-2 (residues 536–923 with an internal deletion of 813–847, Supplementary Figure S1). To understand the structural properties of the NTase region of mammalian GLD-2s, we determined crystal structures of rnGLD-2_131-484_ and mmGLD-2_143-484_ at resolution of 2.5 Å, and 2.7 Å with R*_free_* of 24.3% and 27.1%, respectively (Table 1). Additionally, the two constructs also contain mutations at catalytic sites (D279A for rnGLD-2_131-484_ and D213A/D279A double mutations for mmGLD-2_143-484_), as the yield of wild-type proteins was too low for crystallization experiments. Crystals of rnGLD-2_131-484_ and mmGLD-2_143-484_ both have two independent copies in the asymmetric unit (Supplementary Figure S2A). Superposition of the two rodent structures showed a root mean square deviation (rmsd) of 0.52 Å for 322 aligned Cα atoms (Supplementary Figure S2B). Given this high consistency, we only describe the rnGLD-2_131-484_ structure in the following paragraphs. Unless specified, GLD-2 proteins mentioned in the rest of the paper refer to their NTase regions.

rnGLD-2 is composed of two domains, namely the catalytic domain (from residues 169 to 283) and the central domain (residues 147–168 and 284–480), and shows typical overall architecture of template independent nucleotidyltransferase (Figure 1B). Residues 131–146 and residues 222–230 are disordered and not resolved in the structure. Briefly, the catalytic domain is featured by a five-stranded β-sheet (β1–β5) and two flanking α-helices (α2 and α3). The central domain consists of a seven-α-helical bunch formed by two disconnected parts (helix α1 and helices α4–α9) and a four-stranded β-sheet (β6–β9) (Figure 1C). The two domains embrace a cleft that is sided by the β-sheet of the catalytic domain and helices α5 and α6 of the central domain (Supplementary Figure S3A and B). The cleft harbours the conserved catalytic residues Asp213 and Asp215 on β2, and Asp279 (substituted by an alanine in the structural model) on β5 (Supplementary Figure S3B). Structural comparison with other PAPs containing wild-type catalytic residues indicated that mutation of Asp279 does not affect global or local folding of rnGLD-2 (Supplementary Figure S3C).

### Structural comparison between rnGLD-2 and ceGLD-2

**Figure 2.**
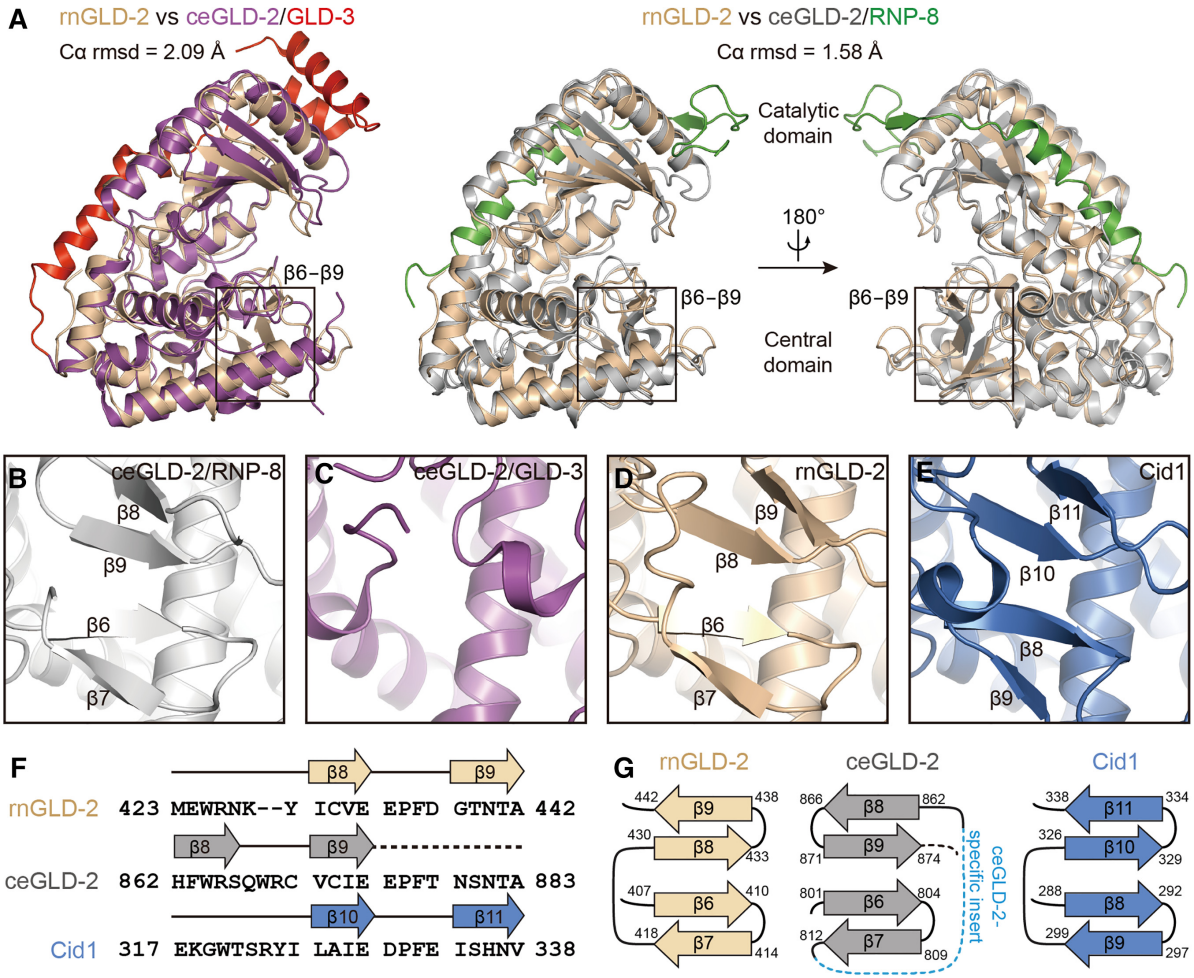
Structure comparison between rnGLD-2 and ceGLD-2. (**A**) Structural superposition of rnGLD-2 with ceGLD-2 complexed with GLD-3 (left, PDB code: 4zrl) or with RNP-8 (right, PDB code: 5jnb). The β6−β9 regions are indicated. (**B** and**C**) Structural details of ceGLD-2 at the tip of the central domain when complexed with RNP-8 (B) or with GLD-3 (C). (**D** and**E**) The β-sheet at the central domain of rnGLD-2 (D) and Cid1 (E). (**F**) Sequence alignment for β8−β9 of GLD-2s from various species and corresponding β10−β11 of Cid1. Residues after β9 of ceGLD-2 that are missing in the structural model are indicated by a dashed line. (**G**) Comparison of the β-sheet at the central domain for rnGLD-2, ceGLD-2 and Cid1 shown as topology diagrams. The ceGLD-2 specific insert is indicated as a blue dashed line. Note the difference in the arrangement of β8 and β9 between rnGLD-2 and ceGLD-2.

The NTase regions of rnGLD-2 and ceGLD-2 share around 32% primary sequence identity and similar architecture. Superposition of rnGLD-2 structure individually with reported ceGLD-2 structures from the ceGLD-2/GLD-3 complex and ceGLD-2/RNP-8 complex yielded rmsd values of 2.09 Å and 1.58 Å for 268 and 288 aligned Cα atoms, respectively (Figure 2A). A major difference of ceGLD-2 in these two structures is seen at the tip of the central domain, the region containing elements of the so-called nucleotide recognition motif (NRM) that determines the nucleotide specificity (20,21). In the ceGLD-2/RNP-8 complex, a four-stranded β-sheet (β6−β9) shields the neighboring helical bunch (Figure 2B), while equivalent region in the ceGLD-2/GLD-3 complex does not form β-sheet (Figure 2C) (20,21). The rnGLD-2 structure we solved possesses a well-folded tip of the central domain where the four-stranded β-sheet is clearly discernable (Figure 2D). However, the two homologs are topologically different at this β-sheet. In rnGLD-2, β9 stays closest to the active site, and the overall topology of the β6−β9 sheet is homologous to the yeast GLD-2-related protein Cid1 (Figure 2E and Supplementary Figure S4A). In the ceGLD-2/RNP-8 complex, the polypeptide of this region winds in a different way. For ceGLD-2, residues 879-NSNTA-883 that are aligned to the sequence of rnGLD-2 β9 are disordered and missing in the structural model, and the corresponding space is taken up by a specific β-strand composed of residues 862-HFWRS-866 (the β8 as defined in reference 21) (Figure 2F and G; Supplementary Figure S1). β8 of rnGLD-2 is aligned to ceGLD-2 β9 in primary and tertiary structure (Figure 2G). This topological variance may be relevant to the ceGLD-2-specific insert at corresponding region (Figure 1A and Supplementary Figure S1), and/or the crystallographic contact involving the N-terminal extension of RNP-8 from a neighboring ceGLD-2/RNP-8 complex (Supplementary Figure S4A).

Like the ceGLD-2 from the ceGLD-2/RNP-8 complex, the catalytic domain and central domain of rnGLD-2 stay closer as compared to ceGLD-2 from the ceGLD-2/GLD-3 complex (Supplementary Figure S4B). For ceGLD-2/RNP-8 complex, this active-like conformation was thought be induced by the crystallographic contact mentioned above (21) (Supplementary Figure S4A). Such contact, however, is not observed in our rnGLD-2 structure (Supplementary Figure S4C), suggesting that rnGLD-2 tends to stay intrinsically in an active-like state even without binding to substrates.

### Mammalian GLD-2s are potent PAPs *in vitro*

**Figure 3.**
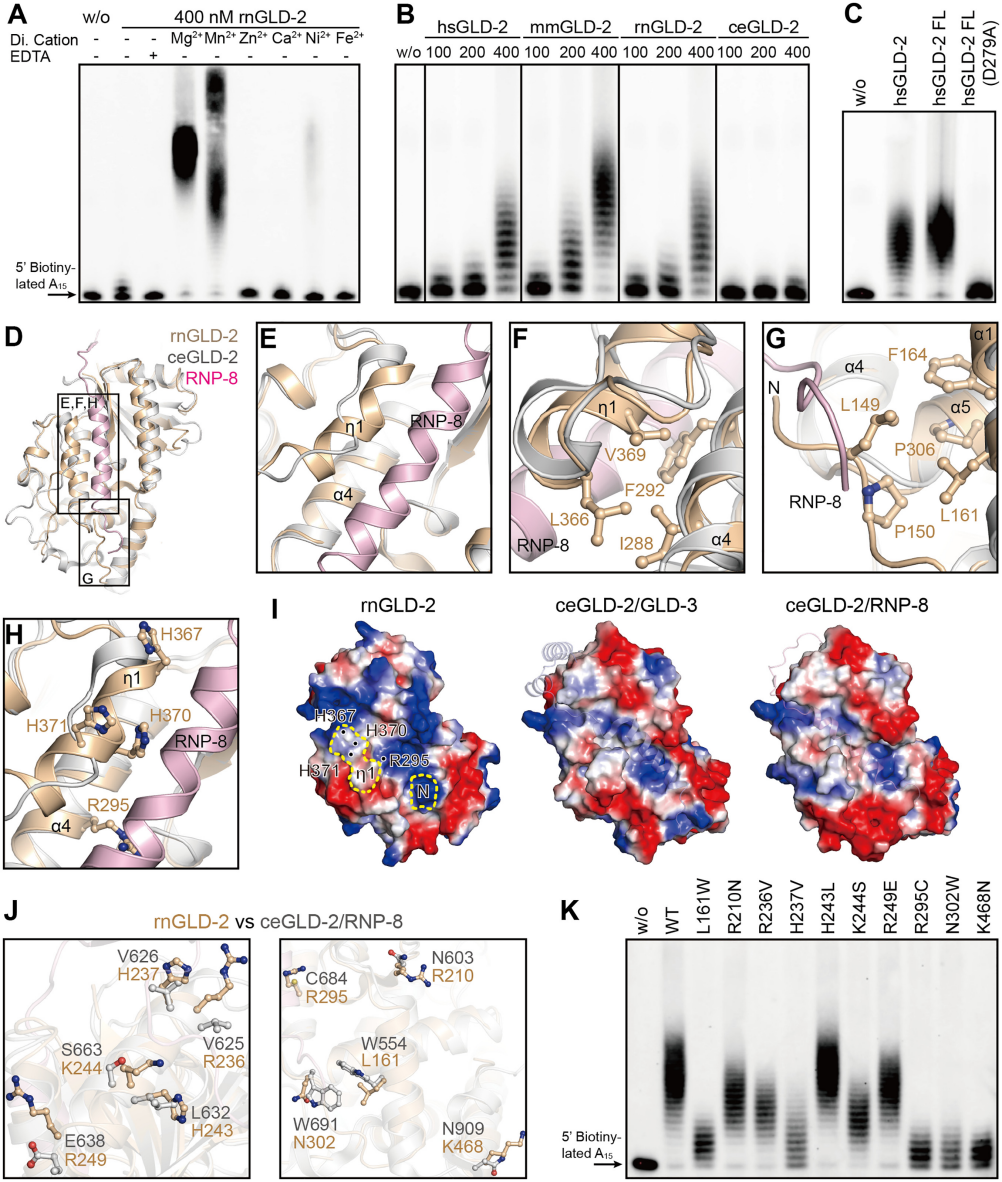
rnGLD-2 is a potent PAP. (**A**) The PAP activity of rnGLD-2 in the presence of various divalent cations. A total of 400 nM rnGLD-2 was incubated with 500 nM 5′ biotinylated A_15_ RNA oligo and 500 μM ATP. For each sample, 2 mM indicated divalent ion or EDTA was supplied to the reaction. w/o, without protein. (**B**) Protein concentration-dependent PAP activity of hsGLD-2, mmGLD-2, rnGLD-2 and ceGLD-2. (**C**) Comparison between truncated and full-length hsGLD-2 in PAP activity. (**D**) Overall structural comparison between rnGLD-2 and ceGLD-2/RNP-8 complex from the backside of the catalytic domain, where the interaction site of ceGLD-2 and RNP-8 can be seen. Frames indicate the areas shown in panels (**E–H**). (E−H) Detailed structure differences between rnGLD-2 and ceGLD-2 explaining the discrepancy of their PAP activity. Color as in D. (E) The unique 3_10_ helix (η1) of rnGLD-2 and its relative position to RNP-8. (F) Interaction between η1 and α4 buries the local hydrophobic cluster of rnGLD-2. (G) The N-terminal tip of rnGLD-2 shelters the hydrophobic residues on α1 and α5. (H) The histidine cluster on η1 and non-conserved Arg295 of rnGLD-2. (**I**) Electrostatic surface potential comparison at the backside of catalytic center for rnGLD-2 (left) and ceGLD-2 complexed with GLD-3 (middle) or RNP-8 (right). Locations of the N-terminal tip (N) and 3_10_ helix (η1) of rnGLD-2 are outlined by yellow dashes, and the histidine cluster and Arg295 are indicated. GLD-3 and RNP-8 are shown in cartoon representation with 50% transparency. (**J**) Non-conserved surface residues between rnGLD-2 and ceGLD-2 that may affect substrate binding. (**K**) PAP activity of rnGLD-2 with mutations regarding the residue difference from ceGLD-2 as shown in (**J**).

To investigate the PAP activity of rnGLD-2, we performed *in vitro* polyadenylation assay using a 5′ biotinylated 15-mer poly(A) RNA substrate (A_15_). The substrate and product RNAs were visualized by means of streptavidin-conjugated chemiluminescence. First, the polyadenylation assay was carried out with the presence of different divalent metal ions. rnGLD-2 was active when magnesium (Mg^2+^) or manganese (Mn^2+^) was supplied (Figure 3A and Supplementary Figure S5A).

Compared to ceGLD-2 which showed very weak PAP activity by itself (20), mammalian GLD-2s efficiently elongated A_15_ substrates in a protein concentration-dependent manner (Figure 3B). Mutations of the consensus NTase catalytic residues abolished the PAP activity of rnGLD-2 (Supplementary Figure S5B). The N-terminal portion (residues 1−130), which is not conserved between mammalian GLD-2s and ceGLD-2, did not significantly influence the PAP activity of human GLD-2 (Figures 1A and 3C). These data indicate that, unlike ceGLD-2 which requires interaction partners to stimulate its PAP activity, mammalian GLD-2s are potent PAPs on their own. For ceGLD-2, association with GLD-3 or RNP-8 is essential for its stability and catalytic activity. According the complex structures, GLD-3 or RNP-8 offers an α-helix lying in an extended groove at the opposite side of ceGLD-2’s catalytic cleft, which shelters the local hydrophobic surface and provides positively charged residues to facilitate binding of the substrate RNA (20,21). At the equivalent position, rnGLD-2 shows several differences from ceGLD-2. First, it has a unique kinked 3_10_ helix (η1) formed by a relatively less conserved region from amino acid residues 366 to 375 in close proximity to α4, whereas corresponding region in ceGLD-2 is not compactly folded (Figure 3D and E; Supplementary Figure S1). Tight association between η1 and α4 of rnGLD-2 buries the side chains of the hydrophobic residues Ile288, Phe292, Leu366 and Val369, and some of the corresponding residues in ceGLD-2 are exposed and require sheltering of GLD-3 or RNP-8 (Figure 3F). Second, the N-terminal tip of rnGLD-2 stretches along a similar direction of the GLD-3 or RNP-8 helix, and covers several hydrophobic residues at the end of the groove (Figure 3G). Third, rnGLD-2 has several non-conserved residues on the surface of the groove, exemplified by Arg295 (corresponding to Cys684 in ceGLD-2) and a specific histidine cluster formed by His367, His370 and His371 on η1 (Figure 3H). These elements take up the binding site for GLD-3/RNP-8 and provide positive charge required for substrate RNA binding (Figure 3I). For the reported ceGLD-2 residues involved in the association with GLD-3 or RNP-8, 10 residues are not conserved between ceGLD-2 and rnGLD-2. While over half of them in ceCLD-2 are hydrophobic, corresponding residues in rnGLD-2 are mostly charged residues according the sequence alignment (Figure 3J and Supplementary Figure S1). We individually mutated these 10 residues on rnGLD-2 to their ceGLD-2 counterparts, and found that the majority of the mutants showed diminished PAP activity (Figure 3K; Supplementary Figure S6A and B). These data explain why mammalian GLD-2s do not need GLD-3- or RNP-8-like partners for the stimulation of their PAP activity.

### Residues important for rnGLD-2’s PAP activity at the catalytic site

**Figure 4.**
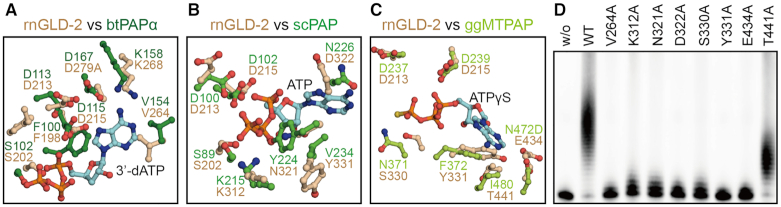
Important residues for ATP coordination of rnGLD-2. (**A−C**) Comparison between rnGLD-2 and other PAPs at adenosine nucleotide binding site. Residues of *Bos taurus* (bt)PAPα (PDB code: 1f5a, A), *Saccharomyces cerevisiae* (sc)PAP (PDB code: 2q66, B) and *Gallus gallus* mitochondria (ggMT)PAP (PDB code: 5a30, C) involved in binding 3′-dATP, ATP and ATPγS are individually superimposed with corresponding residues of rnGLD-2. (**D**) PAP activity of rnGLD-2 with mutations regarding the residues potentially involved in ATP coordination as shown in A−C.

In order to understand how adenosine nucleotide is coordinated by rnGLD-2, we intended to solve the structure of this non-canonical PAP complexed with an adenosine nucleotide. However, ITC experiments showed that rnGLD-2(D279A) lacked the binding affinity to adenosine nucleotides (Supplementary Figure S7), and co-crystallization or soaking did not yield complex structure. Thus, we look for potential ATP-coordinating residues in rnGLD-2 by comparing the active site of rnGLD-2 with those of other eukaryotic PAPs whose complex structure with adenosine nucleotides are available, namely, the canonical *Bos taurus* (bt)PAPα/3′-dATP and *Saccharomyces* cerevisiae(sc)PAP/ATP, and non-canonical *Gallus gallus* mitochondrial (ggMT)PAP/ATP complexes (Figure 4A−C). For these PAPs, the nucleotide substrate specificity and PAP activity are realized by certain residues at the catalytic site (42–44). These residues include the NTase consensus motif (Supplementary Figure S1) and a residue with hydrophobic/aromatic side chain has been found important in stacking the ribose and base moiety of adenosine nucleotides (Phe100 for btPAPα, Val234 for scPAP and Phe372 for ggMTPAP). Their corresponding residues in rnGLD-2 are Phe198 and Tyr331, but Phe198 points away from the nucleotide (Figure 4A−C). On the other hand, these three PAPs each contain specific residues that are also suggested to be important for ATP selectivity. For example, Val154 of btPAPα (corresponding to Val264 of rnGLD-2) is thought to stabilize the adenine moiety of ATP (42), and Asn226 of scPAP (corresponding to Asp322 of rnGLD-2) interacts with the N6 of the adenine ring (43). In addition, Lys312, Asn321 and Ser330 of rnGLD-2 (corresponding to scPAP Lys215, Tyr224 and ggMTPAP Asn371, respectively) may be involved in the contact with phosphate groups and ribose moiety of ATP.

Based on the structural comparison, it is notable that some of the aforementioned residues are not conserved between rnGLD-2 and the three PAPs, which may be a reason for the fact that we failed to obtain stable rnGLD-2-ATP complex for crystallization. We mutated these residues of rnGLD-2 to alanine, and applied these mutants to *in vitro* polyadenylation assay. The result indicated that almost all the mutants showed compromised PAP activity except Thr441 (Figure 4D). This is reasonable because the counterpart of Thr441 in ggMTPAP, Ile480, interacts with the adenine ring of ATP via a hydrogen bond mediated by its main-chain carboxyl group. Thus, it seems that at least some of these residues collectively coordinate ATP in the polyadenylation process.

An interesting point is the possible function of Glu434. Its relevant residue in human MTPAP is Asn478. The mutation of this asparagine in human MTPAP to aspartate (N478D) causes a severe neurodegenerative disease called spastic ataxia 4 (SPAX4), and the poly(A) tails in mitochondrial mRNA of human SPAX4 patients are drastically shortened (45,46). The corresponding N478D mutant of ggMTPAP was shown to be able to bind ATP but lack the PAP activity, and this asparagine was thus proposed to play a role in the positioning of the incoming 3′ nucleotide of substrate mRNA in relation to bound ATP during polyadenylation (44). For rnGLD-2, however, a negatively charged glutamate (Glu434) in this position does not harm its PAP activity, while the mutation of this glutamate to alanine does. This result reflects the importance of this residue for rnGLD-2 and implies a different mechanism of rnGLD-2 in coordinating substrates from other non-classical PAPs.

### rnGLD-2 prefers ATP while showing promiscuity for RNA substrates

**Figure 5.**
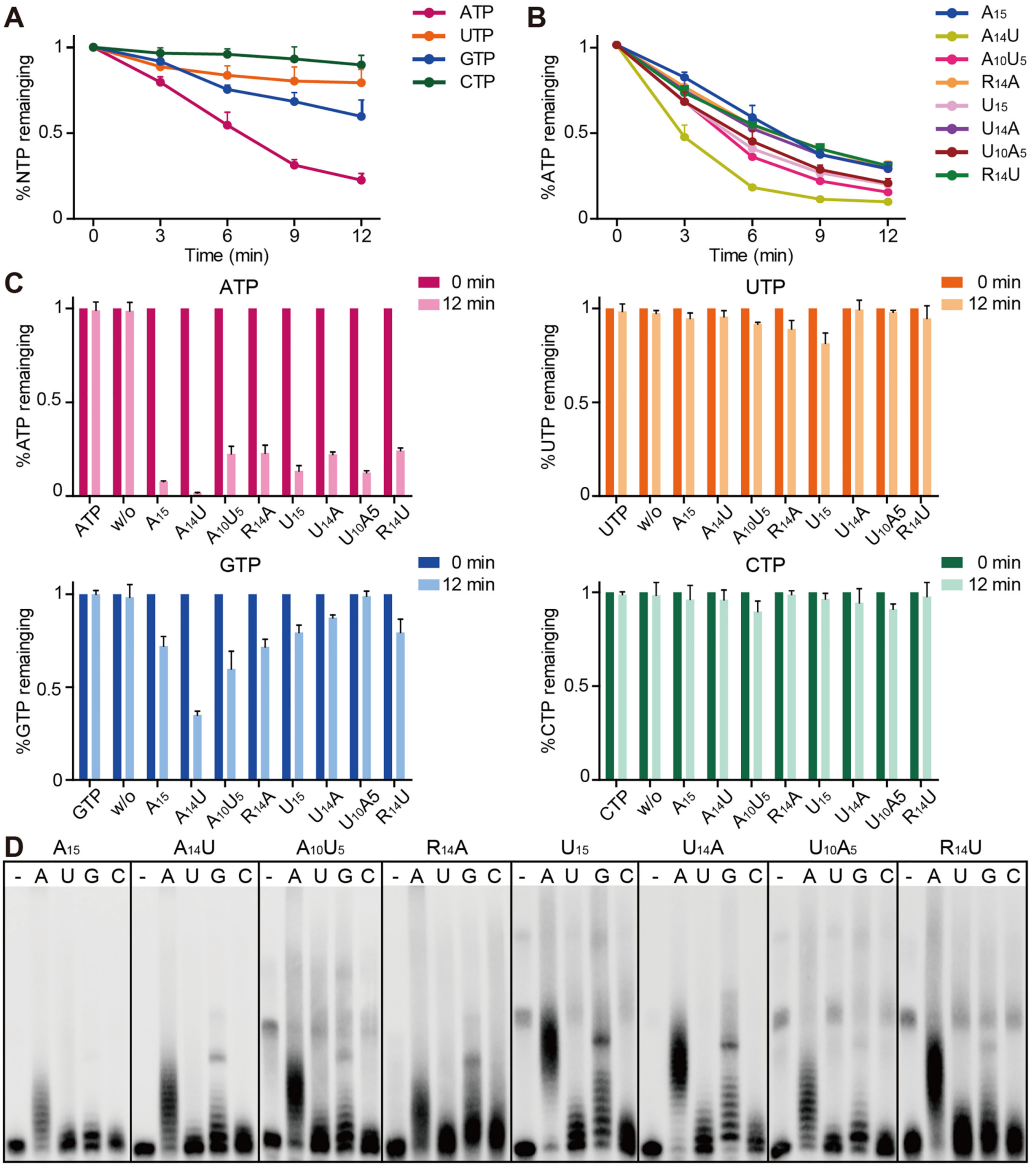
Substrate preference of rnGLD-2. (**A**) Time-course NTP consumption of rnGLD-2 with A_15_. Error bars indicate s.d. (*n* = 3). (**B**) Time-course ATP consumption of rnGLD-2 with various 15-mer RNA oligos. (**C**) NTP consumption of rnGLD-2 with various 15-mer RNA oligos. (**D**) Nucleotidyltransferase assays showing the preference of rnGLD-2 on various substrates and different nucleotides. 400 nM rnGLD-2, 500 nM 5′ biotinylated 15-mer RNA substrates with various sequences, and 500 μM ATP/UTP/GTP/CTP were used.

We next investigated the preference of rnGLD-2 for nucleotides and substrate RNAs. The consumption of ATP/GTP/UTP/CTP in the presence of A_15_ oligo substrate was individually monitored in time course based on an HPLC-based quantification assay (47). After 12 min, around 75% ATP and 40% GTP was consumed by rnGLD-2, whereas over 90% UTP and CTP remained unhydrolyzed, suggesting that rnGLD-2 prefers ATP over other nucleotides during polyadenylation (Figure 5A). Next, 15-mer RNA oligos with different compositions of adenosines and uridines (A_15_, A_14_U, A_10_U_5_, U_15_, U_10_A_5_ and U_14_A), as well as of random nucleotide sequences succeeded by an adenosine or uridine (R_14_A and R_14_U, where R denotes a random nucleotide), were tested in ATP consumption assay. Surprisingly, ATP was substantially consumed in all cases (Figure 5B and C). GTP was also moderately hydrolyzed with most of RNA substrates, while the consumption of UTP and CTP were much less efficient (Figure 5C). Similar results were obtained in the nucleotide incorporation assay, where rnGLD-2 was able to add poly(A) tails to all tested biotinylated 15-mer RNA oligos (Figure 5D). This substrate promiscuity of rnGLD-2 is distinct from the reported substrate preference of ceGLD-2 for adenosine-rich RNA oligos (20).

### rnGLD-2 resembles the catalytic module of TUT7

**Figure 6.**
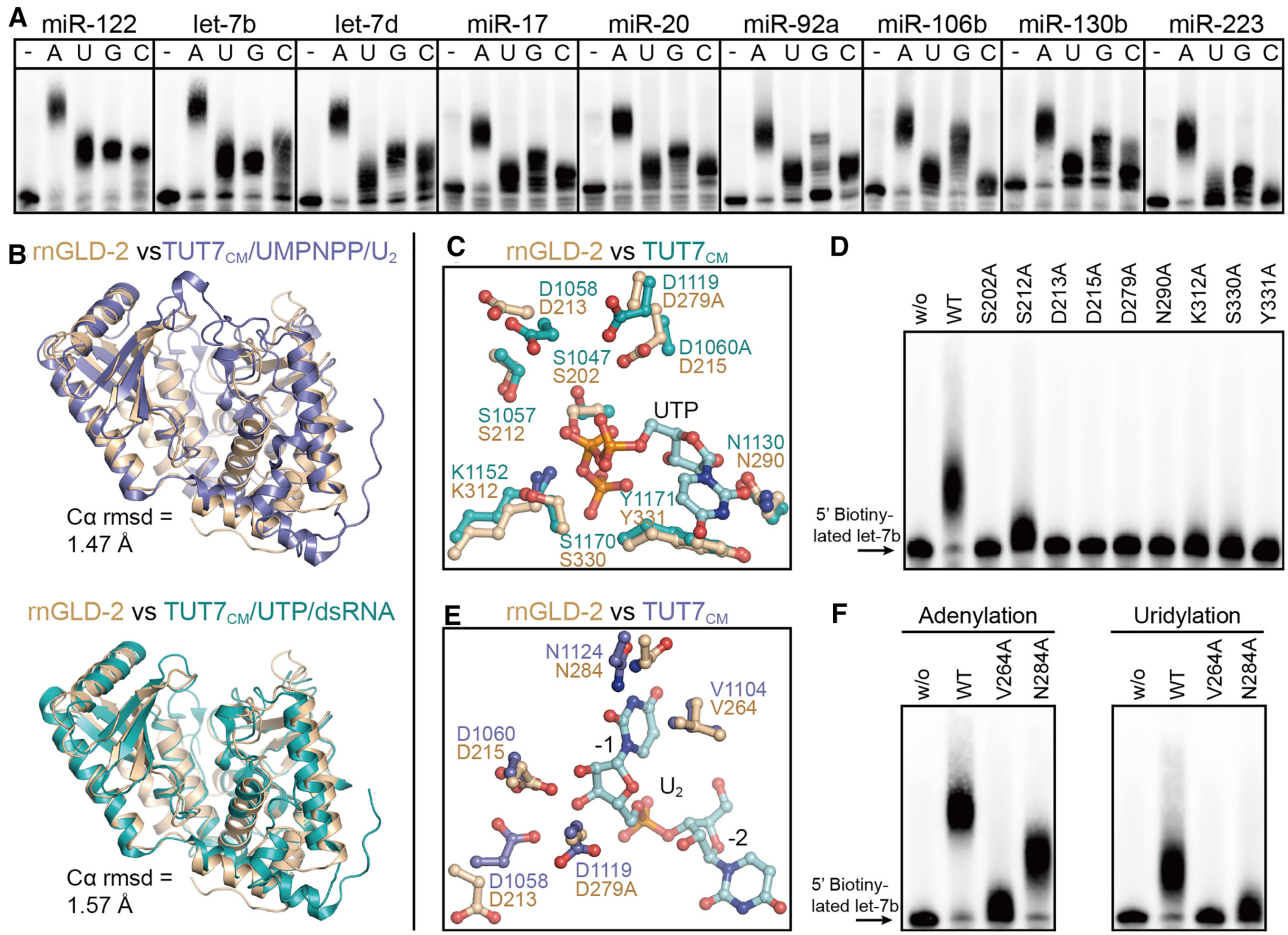
Similarity between rnGLD-2 and TUT7_CM_. (**A**) NTase activity of rnGLD-2 on different miRNA substrates. A total of 400 nM rnGLD-2, 500 nM various 5′-biotinylated miRNA and 500 μM ATP/UTP/GTP/CTP were used. (**B**) Overall structure comparison between rnGLD-2 and TUT7_CM_ in complex with UMPNPP/U_2_ (upper, PDB code: 5w0n) and with UTP/dsRNA (lower, PDB code: 5w0o). (**C**) Comparison between rnGLD-2 and TUT7_CM_ at UTP binding site. Residues of TUT7_CM_ (PDB code: 5w0o) involved in binding UTP is superimposed with corresponding residues of rnGLD-2. (**D**) Uridylation activity of rnGLD-2 with mutations regarding the residues potentially involved in UTP coordination. (**E**) Comparison between rnGLD-2 and TUT7_CM_ at substrate binding site. Residues of TUT7_CM_ (PDB code: 5w0n) involved in binding the U_2_ substrate is superimposed with corresponding residues of rnGLD-2. (**F**) Adenylation and uridylation activity of rnGLD-2 with mutations regarding the residues potentially involved in substrate binding.

Apart from mRNAs, mature miRNAs and pre-miRNAs are also substrates of GLD-2 in mammalian cells (24,28,48). We found that rnGLD-2 was able to extend 3′ tails of various miRNAs and a pre-miRNA *in vitro* (Figure 6A and Supplementary Figure S8). Coincidently, according to the Dali sever (49), the closest structural homolog of rnGLD-2 (except GLD-2 in other species) is the catalytic module (CM) of human terminal uridylyltransferase 7 (TUT7, Supplementary Figure S9), a well-studied miRNA processor. TUT7 are responsible for adding one or multiple uridines to the 3′ end of substrate RNAs, including mRNA, miRNA, and U6 small nuclear RNA (50–52). In particular, TUT7 polyuridylate mRNAs with short poly(A) tails to facilitate global mRNA decay, and mediates the uridylation of some miRNA precursors to control their biogenesis (31,50,53–54). As TUT7 and rnGLD-2 share a similar substrate spectrum, we made a structural comparison between the two proteins to explore the molecular basis of mammalian GLD-2’s miRNA processing activity.

When rnGLD-2 was overlaid to TUT7_CM_ complexed with UMPNPP/U_2_ oligo, or with UTP/double-stranded (ds)RNA, the rmsd values are 1.47 Å or 1.57 Å (Figure 6B). At the nucleotide binding site, rnGLD-2 lacks the consensus UTP-distinguishing histidine possessed by known polyuridylation polymerases (PUPs) such as TUT7 and Cid1, but shares all other UTP coordinating residues with TUT7 (Figure 6C and Supplementary Figure S10A). Mutating either of these conserved residues abolished the uridylation activity of rnGLD-2 on miRNA let-7b (Figure 6D). This is in agreement with the results shown in Figure 6A that rnGLD-2 has uridylation activity but not UTP selectivity.

Next, we sought to understand how rnGLD-2 may bind substrate RNA molecules. In the TUT7_CM_/UMPNPP/U_2_ complex, Asn1124 and Val1104 are involved in docking the uracil base of U_2_ at the −1 position, while the catalytic residues Asp1058 and Asp1060 interact with the ribose of U_2_ (55). These residues are all conserved in rnGLD-2 (Figure 6E). Mutations of Val264 and Asn284 of rnGLD2, which are equivalent to Val1104 and Asn1124 of TUT7, led to diminished adenylation/uridylation activities (Figure 6F). We then investigated the possible interaction between rnGLD-2 and pre-miRNA substrate that contains paired bases by referring to the TUT7_CM_/UTP/dsRNA complex. The overall folding of rnGLD-2 and TUT7_CM_ at the interaction site with the dsRNA substrate are quite similar (Supplementary Figure S10B). In TUT7_CM_, residue Thr1101 and a hydrophobic platform composed of Leu1097 and Leu1099 are crucial in positioning the first base pair of the RNA duplex substrate. rnGLD-2 shows partial conservation with TUT7_CM_ for these elements (Supplementary Figure S10C). Overall, it is possible that mammalian GLD-2 binds miRNA and pre-miRNA substrates in a similar way as TUT7.

### Important surface residues for the NTase activity of rnGLD-2

**Figure 7.**
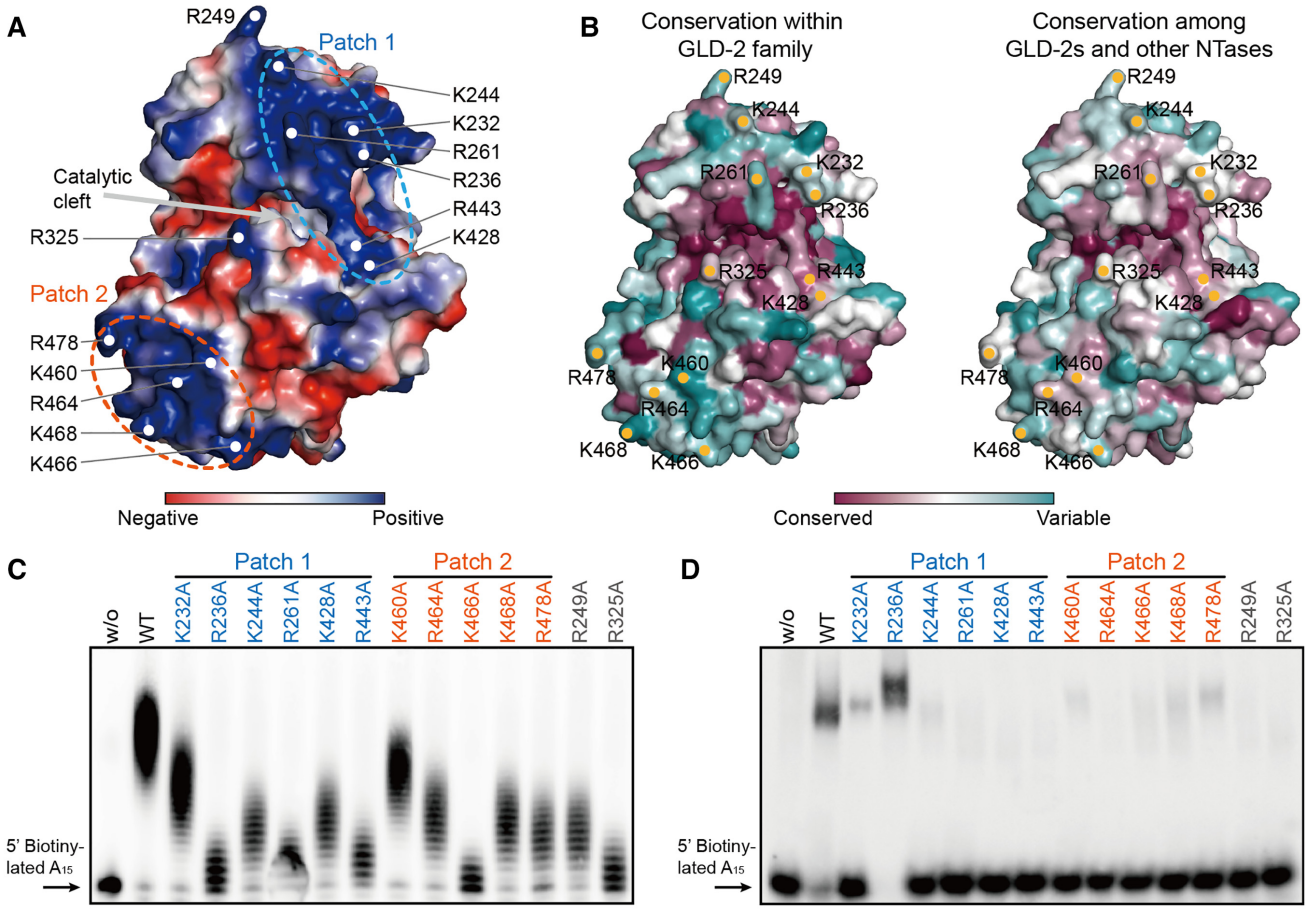
Analysis of positively charged surface residues of rnGLD-2. (**A**) The electrostatic surface potential of rnGLD-2. Some positively charged residues are specified. Two positively charged patches and the catalytic cleft are indicated. (**B**) The surface conservation plots of rnGLD-2 within the GLD-2 family (left) and among other NTases including MTPAP, Cid1, TUT4 and TUT7 (right). (**C**) PAP activity of rnGLD-2 with mutations of positively charged surface residues as in A. (**D**) EMSA results showing the substrate affinity of rnGLD-2 with mutations of positively charged surface residues as in A.

Electrostatic potential plot analysis suggests that rnGLD-2 possesses an extensively charged surface as compared to ceGLD-2. On rnGLD-2, two positively charged patches could be observed on rnGLD-2. Patch 1, formed by Lys232, Arg236, Lys244, Arg261, Lys428 and Arg443, covers the entrance of the catalytic cleft and comprises residues from both catalytic domain and central domain. Patch 2, formed by Lys460, Arg464, Lys466, Lys468 and Arg478, lies at bottom of the central domain relative to the catalytic cleft (Figure 7A). These residues are generally not very conserved among GLD-2s and other NTases including TUTases, Cid1 and mitochondrial PAP (Figure 7B). The most conserved residue is Arg443, which resides on the β6−β9 sheet and faces the incoming RNA substrate (Supplementary Figure S11).

We did mutagenesis analysis on the positively charged residues of the two patches. Compared to wild-type rnGLD-2, the majority of the mutants for patch 1 including Arg443 showed compromised PAP activity, whereas most of the mutants for patch 2 were still potent (Figure 7C and Supplementary Figure S11). Notably, the mutation of Arg325, which is located to the opposite side of patch 1 at the entrance of the catalytic cleft (Figure 7A), severely disrupted the NTase activity of rnGLD-2. Structure comparison between rnGLD-2 and TUT7 revealed that this residue is in close proximity to the RNA substrate, and so are those positively charged residues of patch 1 (Supplementary Figure S11).

To understand whether these residues are directly involved in substrate RNA binding, we performed electrophoretic mobility shift assay (EMSA). rnGLD-2 weakly interacted with A_15_, whereas mutants R325A and R443A showed no binding to A_15_. Mutants that still preserved moderate PAP activities also could associate with A_15_ with a weaker affinity compared to wild-type rnGLD-2 (Figure 7D). An exception was rnGLD-2(R236A), which efficiently bound A_15_ while showing very weak PAP activity. We reasoned that tight association with RNA substrate may hinder the movement of the substrate that is being extended, and hence the successive addition of adenosines. These data indicate that Arg325 and Arg443 of rnGLD-2 contribute to the docking of RNA substrates.

## CONCLUSION

Mammalian GLD-2s are thought to be functionally distinct from their homologs in *C. elegans* and *Xenopus*. While *C. elegans* GLD-2 and *Xenopus* GLD-2 play an indispensable role in the translational control during gametogenesis by polyadenylating certain mRNAs in the cytoplasm (6,15), mice with deletion of *GLD-2* have normal fertility (23). While cytoplasmic polyadenylation in mammalian development is a pivotal event, it is possible that other PAPs take over or share this responsibility in mammals. For example, members of FAM46 proteins, recently reported as active non-canonical PAPs (47,56), showed specifically increased expression during gametogenesis and early embryonic development. The fact that mammalian GLD-2s are intrinsically potent PAPs and stay in a constitutive active state suggests that they may not need GLD-3- or RNP-8-like partners as in *C. elegans* to stimulate PAP activity. This feature of mammalian GLD-2 is in agreement with an *in vivo* cytoplasmic polyadenylation model for *Xenopus* oocytes, where the so-called cytoplasmic polyadenylation complex formed by GLD-2, CPEB, CPSF and symplekin is continuously active, and the inhibition of its activity requires the binding of the poly(A)-specific ribonuclease (PARN), a 3′-deadenylase (57). Other models all include an inhibitory mechanism against a continuously active cytoplasmic polyadenylation complex involving GLD-2 (15).

Apart from the intrinsic PAP activity, the difference between rat GLD-2 and *C. elegans* GLD-2 is also seen for substrate preference. Unlike *C. elegans* GLD-2 which prefers mRNA with poly(A) sequences as substrate *in vitro* (20), we found that rat GLD-2 is active on a variety of substrate RNAs. Similar feature was also reported for human GLD-2 (48). Such difference in substrate preference between *C*. *elegans* and mammalian GLD-2s may result from their topological variance at the β6−β9 sheet of the central domain (Figure 2G). Like in cytoplasmic polyadenylation, the selectivity of GLD-2 on miRNA substrates may be also dependent on the binding with other partners. A recent study suggests that an isoform of QKI7 KH domain-containing RNA binding protein (QKI-7) is responsible for the selective adenylation of miR-122 by GLD-2 in Huh7 cells (58). The adenylation activity of GLD-2 on miR-122 is found to be counterbalanced by deadenylation mediated by CUG-binding protein 1 (CUGBP1) and PARN (59). The plausible versatility of mammalian GLD-2 coincides with its resemblance to TUT7 in structure as well as in substrate spectrum. It seems that evolution has driven GLD-2 toward the modulation of a more complex reservoir of RNAs that reside in mammals (Supplementary Figure S12). Further research is required to pinpoint the exact physiological functions of mammalian GLD-2, for which its communication with specific partners and precise spatiotemporal expression profile need to be taken into account.

## DATA AVAILABILITY

Atomic coordinates and structure factors for the reported crystal structures have been deposited with the Protein Data bank under accession numbers 6LBK (rnGLD-2) and 6LBJ (mmGLD-2).

## Supplementary Material

gkaa578_Supplemental_File

## References

[1] Bardwell V.J., Zarkower D., Edmonds M., Wickens M. The enzyme that adds poly(A) to mRNAs is a classical poly(A) polymerase. Mol. Cell. Biol. 1990; 10:846–849. 2153926 10.1128/mcb.10.2.846-849.1990 PMC360888

[2] Edmonds M. A history of poly A sequences: from formation to factors to function. Prog. Nucleic Acid Res. Mol. Biol. 2002; 71:285–389. 12102557 10.1016/s0079-6603(02)71046-5

[3] Terns M.P., Jacob S.T. Role of poly(A) polymerase in the cleavage and polyadenylation of mRNA precursor. Mol. Cell. Biol. 1989; 9:1435–1444. 2566910 10.1128/mcb.9.4.1435-1444.1989 PMC362560

[4] Schmidt M.J., Norbury C.J. Polyadenylation and beyond: emerging roles for noncanonical poly(A) polymerases. Wiley Interdiscip. Rev. RNA. 2010; 1:142–151. 21956911 10.1002/wrna.16

[5] Laishram R.S., Anderson R.A. The poly A polymerase Star-PAP controls 3′-end cleavage by promoting CPSF interaction and specificity toward the pre-mRNA. EMBO J. 2010; 29:4132–4145. 21102410 10.1038/emboj.2010.287 PMC3018792

[6] Wang L., Eckmann C.R., Kadyk L.C., Wickens M., Kimble J. A regulatory cytoplasmic poly(A) polymerase in Caenorhabditis elegans. Nature. 2002; 419:312–316. 12239571 10.1038/nature01039

[7] Nagaike T., Suzuki T., Katoh T., Ueda T. Human mitochondrial mRNAs are stabilized with polyadenylation regulated by mitochondria-specific poly(A) polymerase and polynucleotide phosphorylase. J. Biol. Chem. 2005; 280:19721–19727. 15769737 10.1074/jbc.M500804200

[8] Cui J., Sackton K.L., Horner V.L., Kumar K.E., Wolfner M.F. Wispy, the Drosophila homolog of GLD-2, is required during oogenesis and egg activation. Genetics. 2008; 178:2017–2029. 18430932 10.1534/genetics.107.084558 PMC2323793

[9] Sartain C.V., Cui J., Meisel R.P., Wolfner M.F. The poly(A) polymerase GLD2 is required for spermatogenesis in Drosophila melanogaster. Development. 2011; 138:1619–1629. 21427144 10.1242/dev.059618 PMC3062429

[10] Rouhana L., Wang L., Buter N., Kwak J.E., Schiltz C.A., Gonzalez T., Kelley A.E., Landry C.F., Wickens M. Vertebrate GLD2 poly(A) polymerases in the germline and the brain. RNA. 2005; 11:1117–1130. 15987818 10.1261/rna.2630205 PMC1370796

[11] Kwak J.E., Wang L., Ballantyne S., Kimble J., Wickens M. Mammalian GLD-2 homologs are poly(A) polymerases. Proc. Natl. Acad. Sci. U.S.A. 2004; 101:4407–4412. 15070731 10.1073/pnas.0400779101 PMC384760

[12] Nousch M., Yeroslaviz A., Eckmann C.R. Stage-specific combinations of opposing poly(A) modifying enzymes guide gene expression during early oogenesis. Nucleic Acids Res. 2019; 47:10881–10893. 31511882 10.1093/nar/gkz787 PMC6845980

[13] Richter J.D., Lasko P. Translational control in oocyte development. Cold Spring Harb. Perspect. Biol. 2011; 3:a002758. 21690213 10.1101/cshperspect.a002758 PMC3181033

[14] Nousch M., Minasaki R., Eckmann C.R. Polyadenylation is the key aspect of GLD-2 function in C. elegans. RNA. 2017; 23:1180–1187. 28490506 10.1261/rna.061473.117 PMC5513063

[15] Radford H.E., Meijer H.A., de Moor C.H. Translational control by cytoplasmic polyadenylation in Xenopus oocytes. Biochim. Biophys. Acta. 2008; 1779:217–229. 18316045 10.1016/j.bbagrm.2008.02.002 PMC2323027

[16] Cui J., Sartain C.V., Pleiss J.A., Wolfner M.F. Cytoplasmic polyadenylation is a major mRNA regulator during oogenesis and egg activation in Drosophila. Dev. Biol. 2013; 383:121–131. 23978535 10.1016/j.ydbio.2013.08.013 PMC3821703

[17] Rissland O.S., Mikulasova A., Norbury C.J. Efficient RNA polyuridylation by noncanonical poly(A) polymerases. Mol. Cell. Biol. 2007; 27:3612–3624. 17353264 10.1128/MCB.02209-06 PMC1899984

[18] Kim K.W., Nykamp K., Suh N., Bachorik J.L., Wang L., Kimble J. Antagonism between GLD-2 binding partners controls gamete sex. Dev. Cell. 2009; 16:723–733. 19460348 10.1016/j.devcel.2009.04.002 PMC2728548

[19] Kim K.W., Wilson T.L., Kimble J. GLD-2/RNP-8 cytoplasmic poly(A) polymerase is a broad-spectrum regulator of the oogenesis program. Proc. Natl. Acad. Sci. U.S.A. 2010; 107:17445–17450. 20855596 10.1073/pnas.1012611107 PMC2951458

[20] Nakel K., Bonneau F., Eckmann C.R., Conti E. Structural basis for the activation of the C. elegans noncanonical cytoplasmic poly(A)-polymerase GLD-2 by GLD-3. Proc. Natl. Acad. Sci. U.S.A. 2015; 112:8614–8619. 26124149 10.1073/pnas.1504648112 PMC4507228

[21] Nakel K., Bonneau F., Basquin C., Habermann B., Eckmann C.R., Conti E. Structural basis for the antagonistic roles of RNP-8 and GLD-3 in GLD-2 poly(A)-polymerase activity. RNA. 2016; 22:1139–1145. 27288313 10.1261/rna.056598.116 PMC4931106

[22] Harris T.W., Arnaboldi V., Cain S., Chan J., Chen W.J., Cho J., Davis P., Gao S., Grove C.A., Kishore R. et al. WormBase: a modern model organism information resource. Nucleic Acids Res. 2020; 48:D762–D767. 31642470 10.1093/nar/gkz920 PMC7145598

[23] Nakanishi T., Kumagai S., Kimura M., Watanabe H., Sakurai T., Kimura M., Kashiwabara S., Baba T. Disruption of mouse poly(A) polymerase mGLD-2 does not alter polyadenylation status in oocytes and somatic cells. Biochem. Biophys. Res. Commun. 2007; 364:14–19. 17927953 10.1016/j.bbrc.2007.09.096

[24] Katoh T., Sakaguchi Y., Miyauchi K., Suzuki T., Kashiwabara S., Baba T., Suzuki T. Selective stabilization of mammalian microRNAs by 3′ adenylation mediated by the cytoplasmic poly(A) polymerase GLD-2. Genes Dev. 2009; 23:433–438. 19240131 10.1101/gad.1761509 PMC2648654

[25] Burns D.M., D’Ambrogio A., Nottrott S., Richter J.D. CPEB and two poly(A) polymerases control miR-122 stability and p53 mRNA translation. Nature. 2011; 473:105–108. 21478871 10.1038/nature09908 PMC3088779

[26] D’Ambrogio A., Gu W., Udagawa T., Mello C.C., Richter J.D. Specific miRNA stabilization by Gld2-catalyzed monoadenylation. Cell Rep. 2012; 2:1537–1545. 23200856 10.1016/j.celrep.2012.10.023 PMC3534910

[27] Peng F., Xiao X., Jiang Y., Luo K., Tian Y., Peng M., Zhang M., Xu Y., Gong G. HBx down-regulated Gld2 plays a critical role in HBV-related dysregulation of miR-122. PLoS One. 2014; 9:e92998. 24667324 10.1371/journal.pone.0092998 PMC3965513

[28] Burroughs A.M., Ando Y., de Hoon M.J., Tomaru Y., Nishibu T., Ukekawa R., Funakoshi T., Kurokawa T., Suzuki H., Hayashizaki Y. et al. A comprehensive survey of 3′ animal miRNA modification events and a possible role for 3′ adenylation in modulating miRNA targeting effectiveness. Genome Res. 2010; 20:1398–1410. 20719920 10.1101/gr.106054.110 PMC2945189

[29] Wyman S.K., Knouf E.C., Parkin R.K., Fritz B.R., Lin D.W., Dennis L.M., Krouse M.A., Webster P.J., Tewari M. Post-transcriptional generation of miRNA variants by multiple nucleotidyl transferases contributes to miRNA transcriptome complexity. Genome Res. 2011; 21:1450–1461. 21813625 10.1101/gr.118059.110 PMC3166830

[30] Heo I., Ha M., Lim J., Yoon M.J., Park J.E., Kwon S.C., Chang H., Kim V.N. Mono-uridylation of pre-microRNA as a key step in the biogenesis of group II let-7 microRNAs. Cell. 2012; 151:521–532. 23063654 10.1016/j.cell.2012.09.022

[31] Kim B., Ha M., Loeff L., Chang H., Simanshu D.K., Li S., Fareh M., Patel D.J., Joo C., Kim V.N. TUT7 controls the fate of precursor microRNAs by using three different uridylation mechanisms. EMBO J. 2015; 34:1801–1815. 25979828 10.15252/embj.201590931 PMC4516432

[32] Wang Q.-S., Zhang K.-H., Cui Y., Wang Z.-J., Pan Q.-Y., Liu K., Sun B., Zhou H., Li M.-J., Xu Q. et al. Upgrade of macromolecular crystallography beamline BL17U1 at SSRF. Nucl. Sci. Tech. 2018; 29:68.

[33] Kabsch W. Xds. Acta Crystallogr. D. Biol. Crystallogr. 2010; 66:125–132. 20124692 10.1107/S0907444909047337 PMC2815665

[34] Keegan R.M., Winn M.D. MrBUMP: an automated pipeline for molecular replacement. Acta Crystallogr. D. Biol. Crystallogr. 2008; 64:119–124. 18094475 10.1107/S0907444907037195 PMC2394800

[35] Winn M.D., Ballard C.C., Cowtan K.D., Dodson E.J., Emsley P., Evans P.R., Keegan R.M., Krissinel E.B., Leslie A.G., McCoy A. et al. Overview of the CCP4 suite and current developments. Acta Crystallogr. D. Biol. Crystallogr. 2011; 67:235–242. 21460441 10.1107/S0907444910045749 PMC3069738

[36] Emsley P., Lohkamp B., Scott W.G., Cowtan K. Features and development of Coot. Acta Crystallogr. D. Biol. Crystallogr. 2010; 66:486–501. 20383002 10.1107/S0907444910007493 PMC2852313

[37] Adams P.D., Afonine P.V., Bunkoczi G., Chen V.B., Davis I.W., Echols N., Headd J.J., Hung L.W., Kapral G.J., Grosse-Kunstleve R.W. et al. PHENIX: a comprehensive Python-based system for macromolecular structure solution. Acta Crystallogr. D. Biol. Crystallogr. 2010; 66:213–221. 20124702 10.1107/S0907444909052925 PMC2815670

[38] Chen V.B., Arendall W.B. 3rd, Headd J.J., Keedy D.A., Immormino R.M., Kapral G.J., Murray L.W., Richardson J.S., Richardson D.C. MolProbity: all-atom structure validation for macromolecular crystallography. Acta Crystallogr. D. Biol. Crystallogr. 2010; 66:12–21. 20057044 10.1107/S0907444909042073 PMC2803126

[39] UniProt C. UniProt: a worldwide hub of protein knowledge. Nucleic Acids Res. 2019; 47:D506–D515. 30395287 10.1093/nar/gky1049 PMC6323992

[40] Madeira F., Park Y.M., Lee J., Buso N., Gur T., Madhusoodanan N., Basutkar P., Tivey A.R.N., Potter S.C., Finn R.D. et al. The EMBL-EBI search and sequence analysis tools APIs in 2019. Nucleic Acids Res. 2019; 47:W636–W641. 30976793 10.1093/nar/gkz268 PMC6602479

[41] Ashkenazy H., Abadi S., Martz E., Chay O., Mayrose I., Pupko T., Ben-Tal N. ConSurf 2016: an improved methodology to estimate and visualize evolutionary conservation in macromolecules. Nucleic Acids Res. 2016; 44:W344–W350. 27166375 10.1093/nar/gkw408 PMC4987940

[42] Martin G., Keller W., Doublie S. Crystal structure of mammalian poly(A) polymerase in complex with an analog of ATP. EMBO J. 2000; 19:4193–4203. 10944102 10.1093/emboj/19.16.4193 PMC302044

[43] Balbo P.B., Bohm A. Mechanism of poly(A) polymerase: structure of the enzyme-MgATP-RNA ternary complex and kinetic analysis. Structure. 2007; 15:1117–1131. 17850751 10.1016/j.str.2007.07.010 PMC2032019

[44] Lapkouski M., Hallberg B.M. Structure of mitochondrial poly(A) RNA polymerase reveals the structural basis for dimerization, ATP selectivity and the SPAX4 disease phenotype. Nucleic Acids Res. 2015; 43:9065–9075. 26319014 10.1093/nar/gkv861 PMC4605311

[45] Crosby A.H., Patel H., Chioza B.A., Proukakis C., Gurtz K., Patton M.A., Sharifi R., Harlalka G., Simpson M.A., Dick K. et al. Defective mitochondrial mRNA maturation is associated with spastic ataxia. Am. J. Hum. Genet. 2010; 87:655–660. 20970105 10.1016/j.ajhg.2010.09.013 PMC2978972

[46] Wilson W.C., Hornig-Do H.T., Bruni F., Chang J.H., Jourdain A.A., Martinou J.C., Falkenberg M., Spahr H., Larsson N.G., Lewis R.J. et al. A human mitochondrial poly(A) polymerase mutation reveals the complexities of post-transcriptional mitochondrial gene expression. Hum. Mol. Genet. 2014; 23:6345–6355. 25008111 10.1093/hmg/ddu352 PMC4222368

[47] Hu J.L., Liang H., Zhang H., Yang M.Z., Sun W., Zhang P., Luo L., Feng J.X., Bai H., Liu F. et al. FAM46B is a prokaryotic-like cytoplasmic poly(A) polymerase essential in human embryonic stem cells. Nucleic Acids Res. 2020; 48:2733–2748. 32009146 10.1093/nar/gkaa049 PMC7049688

[48] Chung C.Z., Jo D.H., Heinemann I.U. Nucleotide specificity of the human terminal nucleotidyltransferase Gld2 (TUT2). RNA. 2016; 22:1239–1249. 27284165 10.1261/rna.056077.116 PMC4931116

[49] Holm L. Benchmarking Fold Detection by DaliLite v.5. Bioinformatics. 2019; 35:5326–5327. 31263867 10.1093/bioinformatics/btz536

[50] Lim J., Ha M., Chang H., Kwon S.C., Simanshu D.K., Patel D.J., Kim V.N. Uridylation by TUT4 and TUT7 marks mRNA for degradation. Cell. 2014; 159:1365–1376. 25480299 10.1016/j.cell.2014.10.055 PMC4720960

[51] Thornton J.E., Chang H.M., Piskounova E., Gregory R.I. Lin28-mediated control of let-7 microRNA expression by alternative TUTases Zcchc11 (TUT4) and Zcchc6 (TUT7). RNA. 2012; 18:1875–1885. 22898984 10.1261/rna.034538.112 PMC3446710

[52] Trippe R., Guschina E., Hossbach M., Urlaub H., Luhrmann R., Benecke B.J. Identification, cloning, and functional analysis of the human U6 snRNA-specific terminal uridylyl transferase. RNA. 2006; 12:1494–1504. 16790842 10.1261/rna.87706 PMC1524887

[53] Heo I., Joo C., Kim Y.K., Ha M., Yoon M.J., Cho J., Yeom K.H., Han J., Kim V.N. TUT4 in concert with Lin28 suppresses microRNA biogenesis through pre-microRNA uridylation. Cell. 2009; 138:696–708. 19703396 10.1016/j.cell.2009.08.002

[54] Morgan M., Much C., DiGiacomo M., Azzi C., Ivanova I., Vitsios D.M., Pistolic J., Collier P., Moreira P.N., Benes V. et al. mRNA 3′ uridylation and poly(A) tail length sculpt the mammalian maternal transcriptome. Nature. 2017; 548:347–351. 28792939 10.1038/nature23318 PMC5768236

[55] Faehnle C.R., Walleshauser J., Joshua-Tor L. Multi-domain utilization by TUT4 and TUT7 in control of let-7 biogenesis. Nat. Struct. Mol. Biol. 2017; 24:658–665. 28671666 10.1038/nsmb.3428 PMC5542866

[56] Kuchta K., Muszewska A., Knizewski L., Steczkiewicz K., Wyrwicz L.S., Pawlowski K., Rychlewski L., Ginalski K. FAM46 proteins are novel eukaryotic non-canonical poly(A) polymerases. Nucleic Acids Res. 2016; 44:3534–3548. 27060136 10.1093/nar/gkw222 PMC4857005

[57] Kim J.H., Richter J.D. Opposing polymerase-deadenylase activities regulate cytoplasmic polyadenylation. Mol. Cell. 2006; 24:173–183. 17052452 10.1016/j.molcel.2006.08.016

[58] Hojo H., Yashiro Y., Noda Y., Ogami K., Yamagishi R., Okada S., Hoshino S.I., Suzuki T. The RNA-binding protein QKI-7 recruits the poly(A) polymerase GLD-2 for 3′ adenylation and selective stabilization of microRNA-122. J. Biol. Chem. 2020; 295:390–402. 31792053 10.1074/jbc.RA119.011617 PMC6956528

[59] Katoh T., Hojo H., Suzuki T. Destabilization of microRNAs in human cells by 3′ deadenylation mediated by PARN and CUGBP1. Nucleic Acids Res. 2015; 43:7521–7534. 26130707 10.1093/nar/gkv669 PMC4551920

