# Supplementary material for: Structures of mammalian GLD-2 proteins reveal molecular basis of their functional diversity in mRNA and microRNA processing
Source: Nucleic Acids Res. 2020 Jul 7;48(15):8782–95. doi: 10.1093/nar/gkaa578 (PMC7470959; doi:10.1093/nar/gkaa578)
Supplement: gkaa578_Supplemental_File [file gkaa578_supplemental_file.pdf]

Supplementary Material

Structures of mammalian GLD-2 proteins reveal molecular basis of their functional diversity in mRNA and microRNA processing

Xiao-Yan Ma[1,†], Hong Zhang[1,†], Jian-Xiong Feng[1], Jia-Li Hu[1], Bing Yu[1], Li Luo[1], Yu-Lu Cao[1], Shuang Liao[1], Jichang Wang[2,3] and Song Gao[1,4]*

Affiliations

1. State Key Laboratory of Oncology in South China, Collaborative Innovation Center for Cancer Medicine, Sun Yat-sen University Cancer Center, Guangzhou, Guangdong, 510060, China

2. Key Laboratory for Stem Cells and Tissue Engineering (Sun Yat-Sen University), Ministry of Education, Guangzhou 510080, China

3. Department of histology and embryology, Zhongshan School of Medicine, Sun Yat-sen University, Guangzhou 510080, China

4. Guangzhou Regenerative Medicine and Health Guangdong Laboratory, Guangzhou 510530, China.

* To whom correspondence should be addressed. Tel: +86-20-87343168; Fax: +86-20-87343170; Email: gaosong@sysucc.org.cn.

† The authors wish it to be known that, in their opinion, the first two authors should be regarded as Joint First Authors.

Supplementary Figures

Figure S1: Sequence alignment for the NTase region of GLD-2s

Figure S2: Structure comparison between rnGLD-2 and mmGLD-2

Figure S3: The activity center of rnGLD2

Figure S4: Structure of rnGLD-2 represents an active-like state

Figure S5: PAP activity of mammalian GLD-2s

Figure S6: Properties of charged-to-hydrophobic mutants of rnGLD-2

Figure S7: rnGLD-2 lacks binding affinity to adenosine nucleotides

Figure S8: rnGLD-2 shows uridylation activity on pre-let-7a

Figure S9: Results of Dali search for rnGLD-2, mmGLD-2, and ceGLD-2

Figure S10: rnGLD-2 structurally resembles $TUT7_{CM}$

Figure S11: Positively charged surface residues of rnGLD-2 potentially involved in substrate binding

Figure S12: Summary of the cellular function of mammalian GLD-2

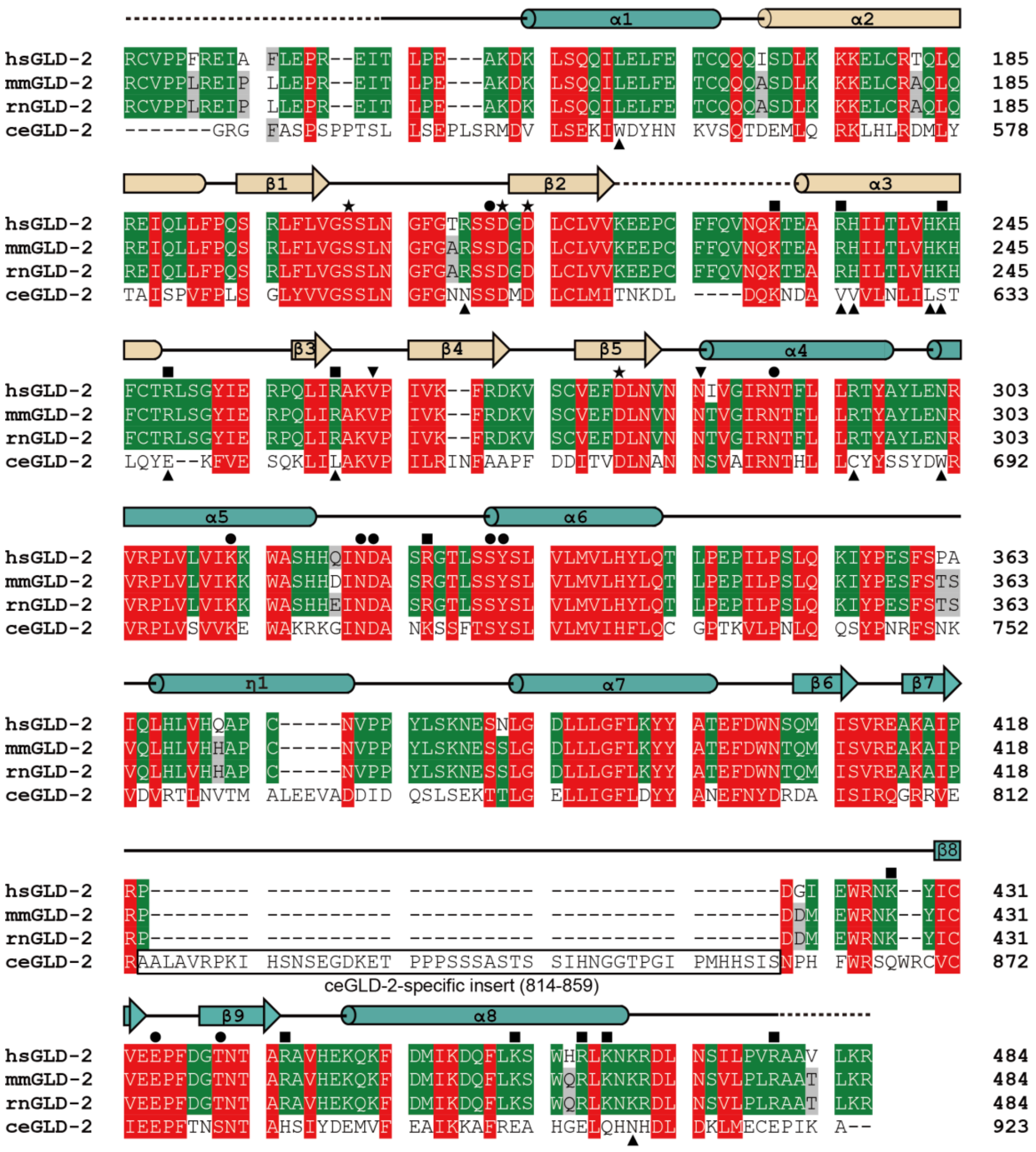

★ Consensus NTase catalytic residues ▲ Nonconserved surface residues between rnGLD-2 and ceGLD-2

● Putative nucleotide coordinating residues ▼ Putative RNA substrate binding sites

■ Positively charged surface residues that may be involoved in substrate binding

Figure S1, Sequence alignment for the NTase region of GLD-2s

Amino acid sequences of human (hs)GLD-2 (UniProt accession Q6PIY7), mouse (mm)GLD-2 (Q91YI6), rat (rn)GLD-2 (Q5U315) and *C. elegans* (ce)GLD-2 (O17089) are aligned. Residues with a conservation of 100% are in red shades, greater than 70% in green shades and 45% in grey shades, respectively. α-helices are shown as cylinders and β-strands as arrows for rnGLD-2 above the sequences. The secondary structure signs are coloured and labelled as in Fig. **1B**. Regions not resolved in the crystal structure are indicated by dashed lines. Important residues that are discussed in the paper are specified by different symbols according to their functional roles.

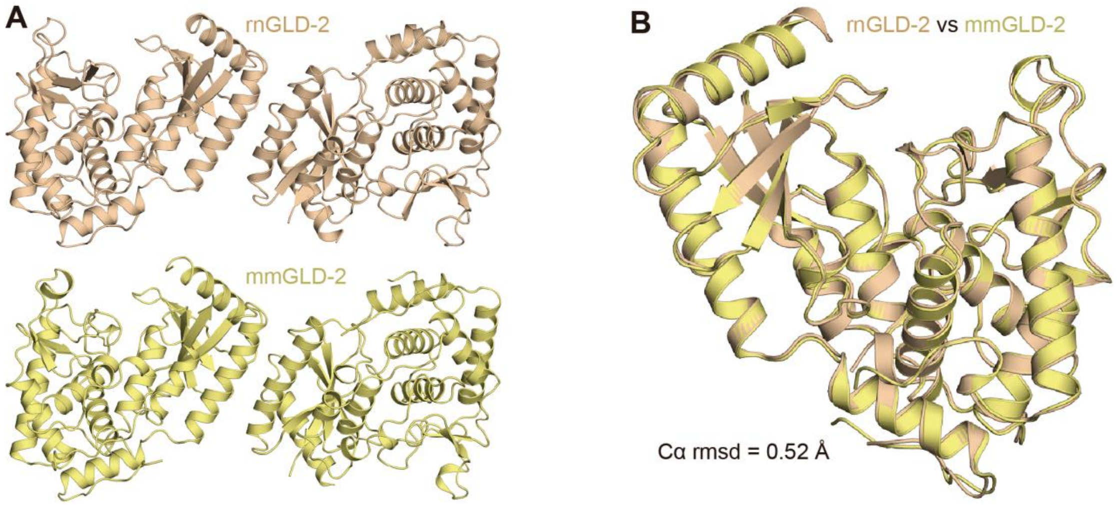

Figure S2, Structure comparison between rnGLD-2 and mmGLD-2

(A) The crystallographic dimers of rnGLD2 and mmGLD2 in the minimum asymmetric unit. rnGLD-2 and mmGLD-2 are coloured wheat and pale yellow, respectively.

(B) Superposition of rnGLD-2 and mmGLD-2. Colour as in **A**.

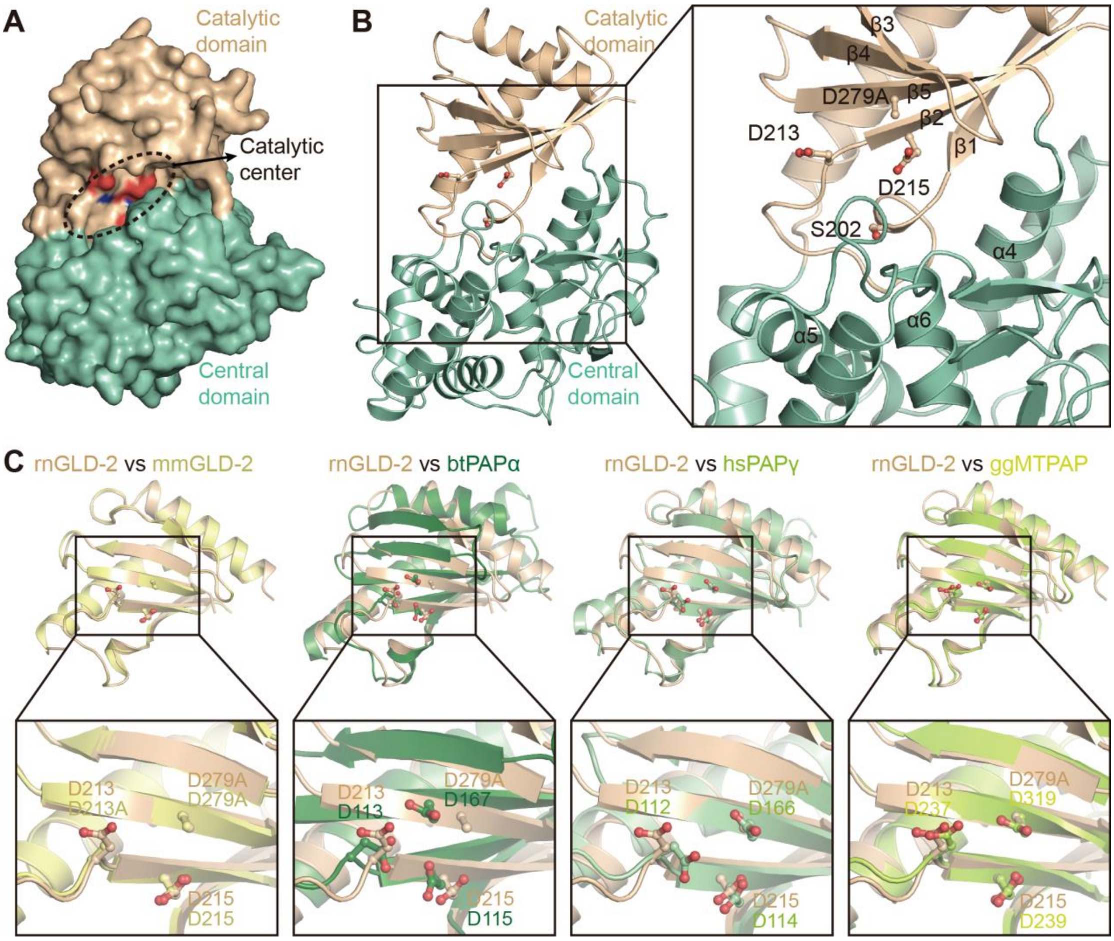

Figure S3, The activity center of rnGLD2

(A) Surface representation of the cleft between the catalytic domain and central domain as indicated by dashed ellipse. The catalytic residues are coloured red.

(B) The consensus NTase motif in rnGLD-2. The key catalytic residues are indicated as ball-and-stick models, and secondary structural elements surrounding the catalytic center are specified.

(C) Structure comparison of rnGLD-2 with mmGLD-2 and other PAPs (btPAPα: PDB code 1f5a; hsPAPγ: 4lt6; ggMTPAP: 5a30) with native catalytic residues showing that the D279A mutation of rnGLD-2 does not affect the overall and local folding of rnGLD-2. The catalytic domain (upper) and catalytic site (lower) of rnGLD-2 is individually superimposed.

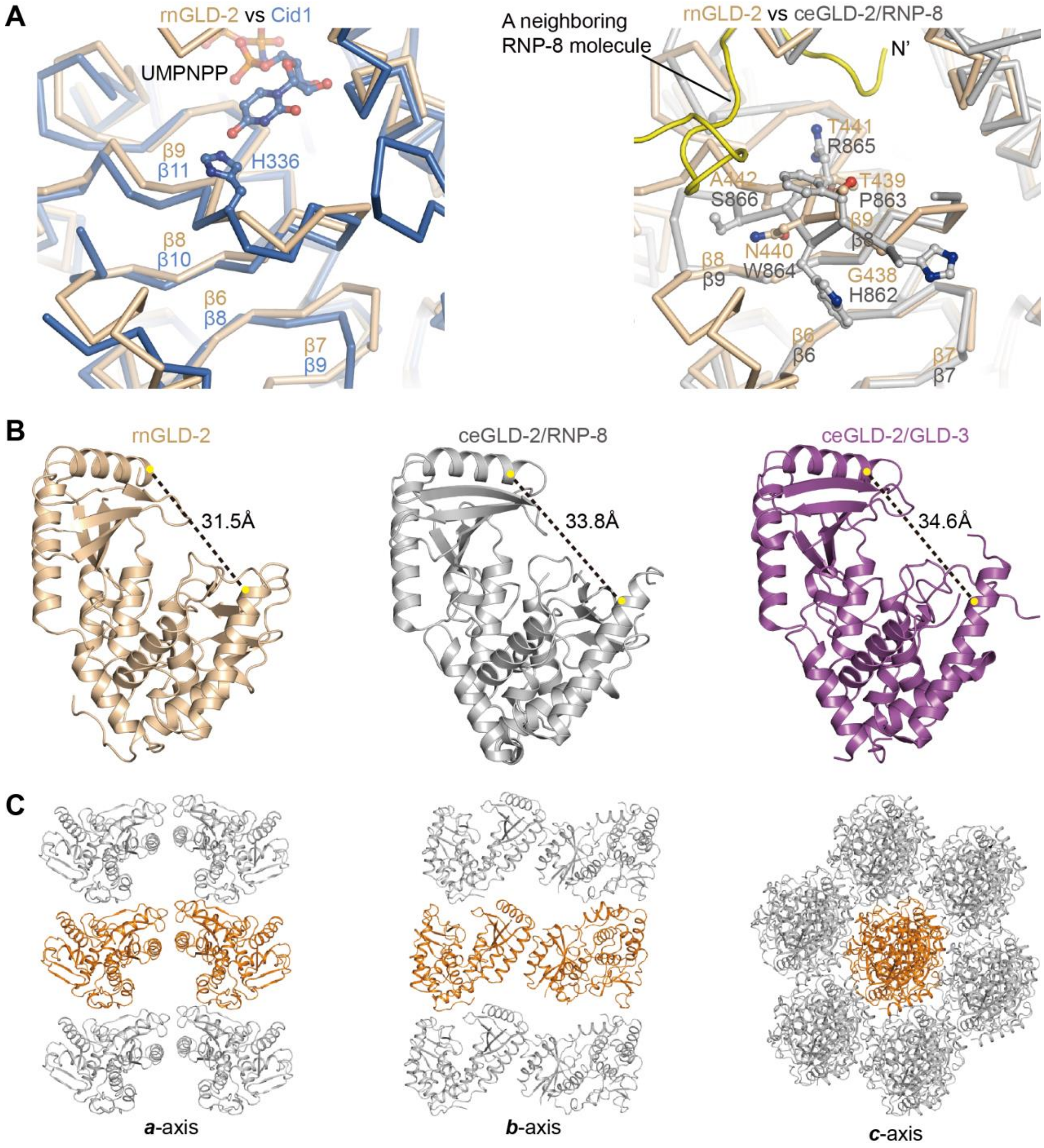

Figure S4, Structure of rnGLD-2 represents an active-like state

(A) Structural comparisons between the β6–β9 region in rnGLD-2 and equivalent regions in Cid1 (complexed with UMPNPP, left) and in ceGLD-2 (complexed with RNP-8, right). In left panel, the UTP-selecting histidine (His336) is shown as ball-and-stick models. In right panel, residues of rnGLD-2 β9 and ceGLD-2 β9 neighbouring the nucleotide binding pocket are shown as ball-and-stick models for comparison. Note that the N-terminal portion of a RNP-8 molecule from the neighbouring asymmetric unit of the crystal (coloured yellow) inserts into the active site of ceGLD-2.

(B) rnGLD-2 showed a more closed conformation as compared to ceGLD-2, as indicated by the width of the cleft between the catalytic and central domains. The width is defined by the distance between the Cα atoms of residues Arg236 and Lys450 in rnGLD-2, and of their corresponding residues Val625 and Val891 in ceGLD-2.

(C) Crystal packing of rnGLD-2 viewed along *a*, *b* and *c*-axes of the unit cell. Two rnGLD-2 molecules (a copy of which is coloured orange) are in the asymmetric unit of the crystal.

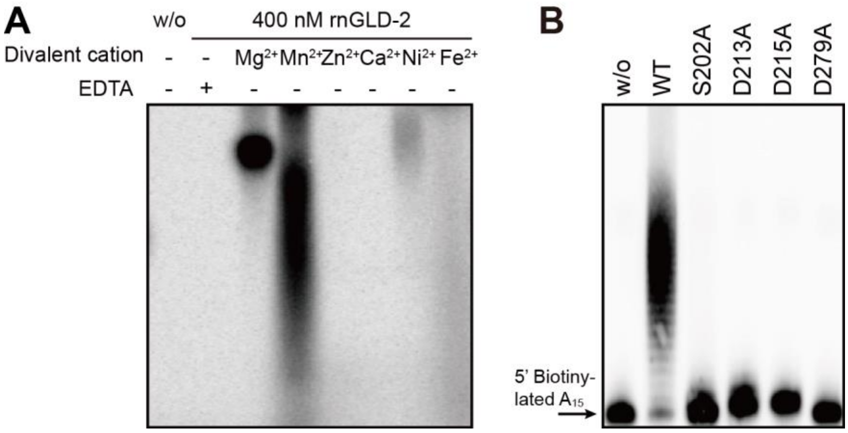

Figure S5, PAP activity of mammalian GLD-2s

(A) PAP activity of rnGLD-2 (400 nM) in the presence of different divalent ions (2 mM) indicated by the incorporation of ATP (300 μM) and 12 μCi/mmol ^{32}P labelled ATP to the A_{15} (500 nM) substrate. w/o, without protein.

(B) Mutation of the consensus NTase catalytic residues abolishes the PAP activity of rnGLD-2. 400 nM wild-type (WT) rnGLD-2 or mutants were used.

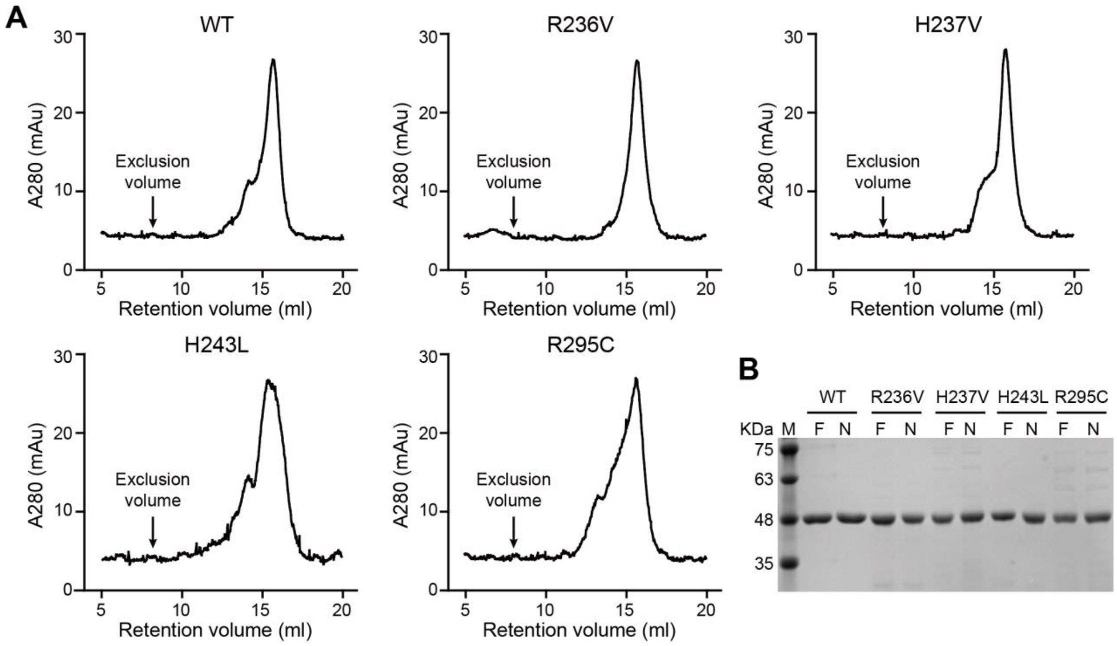

Figure S6, Properties of charged-to-hydrophobic mutants of rnGLD-2

(A) Analytical gel filtration results for the rnGLD-2 mutants showing that they do not aggregate in solution. The exclusion volume of the column is indicated for each chromatograph, where no peak for aggregating protein is seen.

(B) SDS-PAGE analysis of the rnGLD-2 mutants showing that they do not degrade. For each protein, the freshly (F) purified sample and the sample after 4°C incubation overnight (N) are analysed in parallel. M, marker for molecular weight.

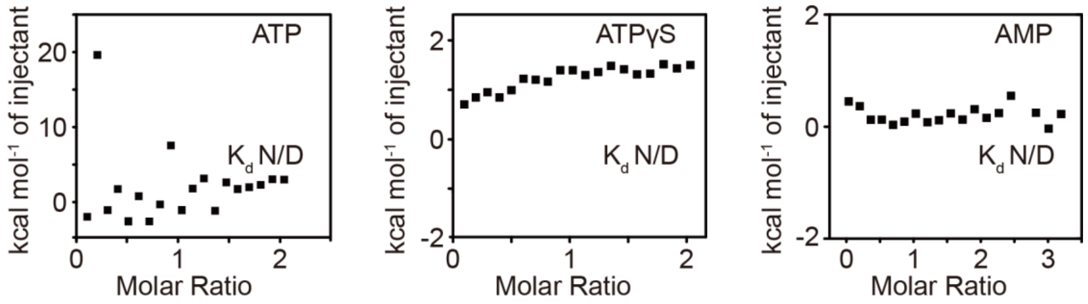

Figure S7, rnGLD-2 lacks binding affinity to adenosine nucleotides

Binding affinities (dissociation constant, Kd) between rnGLD-2(D279A) and ATP, ATPγS, or AMP were measured by isothermal titration calorimetry (ITC). rnGLD-2(D279A) lacks binding affinity to all these nucleotides. N/D, not determined.

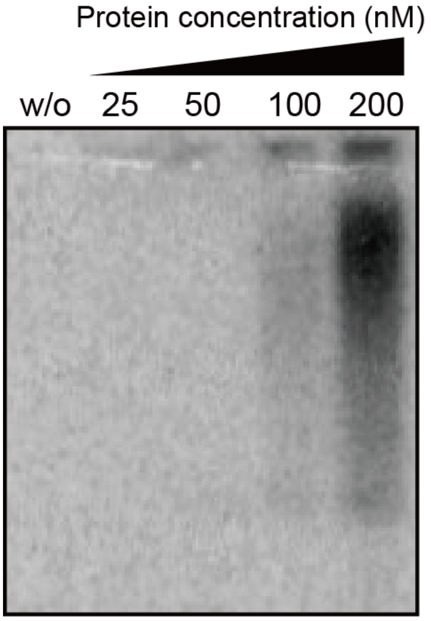

Figure S8, rnGLD-2 shows uridylation activity on pre-let-7a

Uridylation activity of rnGLD-2 on the precursor miRNA pre-let-7a (500 nM) at different protein concentrations, indicated by the incorporation of UTP (300 μM) and 12 μCi/mmol ^{32}P labelled UTP.

Query: 6lbkA (rnGLD-2)

No*	Chain	Z	rmsd	lali	nres	%id	PDB Description	
1:	5jnb-B	35.7	1.8	296	324	40	MOLECULE: POLY(A) RNA POLYMERASE GLD-2;	(ceGLD-2)
2:	5jnb-A	35.3	1.8	294	329	41	MOLECULE: POLY(A) RNA POLYMERASE GLD-2;	(ceGLD-2)
3:	5w0n-C	34.2	2.1	296	350	31	MOLECULE: TERMINAL URIDYLYLTRANSFERASE 7;	(hsTUT7)
4:	5w0m-B	34.1	2.0	295	350	31	MOLECULE: TERMINAL URIDYLYLTRANSFERASE 7;	(hsTUT7)
5:	5w0o-A	34.1	2.2	295	338	30	MOLECULE: TERMINAL URIDYLYLTRANSFERASE 7;	(hsTUT7)
6:	5w0m-C	34.0	2.0	295	371	31	MOLECULE: TERMINAL URIDYLYLTRANSFERASE 7;	(hsTUT7)
7:	5w0n-B	34.0	2.0	295	372	31	MOLECULE: TERMINAL URIDYLYLTRANSFERASE 7;	(hsTUT7)
8:	5a2w-B	34.0	2.2	298	467	25	MOLECULE: MITOCHONDRIAL PROTEIN;	(ggMTPAP)
9:	5w0n-A	33.8	2.0	295	379	31	MOLECULE: TERMINAL URIDYLYLTRANSFERASE 7;	(hsTUT7)
10:	5w0m-A	33.8	2.1	296	372	31	MOLECULE: TERMINAL URIDYLYLTRANSFERASE 7;	(hsTUT7)

Query: 6lbjA (mmGLD-2)

No*	Chain	Z	rmsd	lali	nres	%id	PDB Description	
1:	5jnb-B	35.4	1.8	293	324	40	MOLECULE: POLY(A) RNA POLYMERASE GLD-2;	(ceGLD-2)
2:	5jnb-A	34.9	1.8	291	329	40	MOLECULE: POLY(A) RNA POLYMERASE GLD-2;	(ceGLD-2)
3:	5w0n-C	34.1	2.2	298	350	31	MOLECULE: TERMINAL URIDYLYLTRANSFERASE 7;	(hsTUT7)
4:	5w0m-B	34.0	2.1	296	350	31	MOLECULE: TERMINAL URIDYLYLTRANSFERASE 7;	(hsTUT7)
5:	5w0o-A	33.9	2.1	293	338	30	MOLECULE: TERMINAL URIDYLYLTRANSFERASE 7;	(hsTUT7)
6:	5w0n-B	33.8	2.2	297	372	31	MOLECULE: TERMINAL URIDYLYLTRANSFERASE 7;	(hsTUT7)
7:	5w0m-C	33.8	2.2	297	371	31	MOLECULE: TERMINAL URIDYLYLTRANSFERASE 7;	(hsTUT7)
8:	5w0n-A	33.6	2.2	298	379	31	MOLECULE: TERMINAL URIDYLYLTRANSFERASE 7;	(hsTUT7)
9:	5w0m-A	33.6	2.2	298	372	31	MOLECULE: TERMINAL URIDYLYLTRANSFERASE 7;	(hsTUT7)
10:	5jnb-C	33.5	1.7	276	289	41	MOLECULE: POLY(A) RNA POLYMERASE GLD-2;	(ceGLD-2)

Query: 5jnbA (ceGLD-2/RNP8)

No*	Chain	Z	rmsd	lali	nres	%id	PDB Description	
1:	5jnb-A	54.8	0.0	329	329	100	MOLECULE: POLY(A) RNA POLYMERASE GLD-2;	(ceGLD-2)
2:	5jnb-B	50.9	0.4	323	324	99	MOLECULE: POLY(A) RNA POLYMERASE GLD-2;	(ceGLD-2)
3:	5jnb-C	40.9	0.8	289	289	100	MOLECULE: POLY(A) RNA POLYMERASE GLD-2;	(ceGLD-2)
4:	4zrl-A	36.1	2.1	300	313	88	MOLECULE: POLY(A) RNA POLYMERASE GLD-2;	(ceGLD-2)
5:	5jnb-D	35.9	0.7	263	263	100	MOLECULE: POLY(A) RNA POLYMERASE GLD-2;	(ceGLD-2)
6:	5w0m-C	31.0	2.3	297	371	28	MOLECULE: TERMINAL URIDYLYLTRANSFERASE 7;	(hsTUT7)
7:	5w0m-A	30.9	2.4	299	372	28	MOLECULE: TERMINAL URIDYLYLTRANSFERASE 7;	(hsTUT7)
8:	5w0m-B	30.8	2.4	297	350	29	MOLECULE: TERMINAL URIDYLYLTRANSFERASE 7;	(hsTUT7)
9:	5w0n-C	30.8	2.4	296	350	29	MOLECULE: TERMINAL URIDYLYLTRANSFERASE 7;	(hsTUT7)
10:	5w0n-B	30.8	2.4	297	372	28	MOLECULE: TERMINAL URIDYLYLTRANSFERASE 7;	(hsTUT7)

Query: 4zrlA (ceGLD-2/GLD-3)

No*	Chain	Z	rmsd	lali	nres	%id	PDB Description	
1:	4zrl-A	52.2	0.0	313	313	100	MOLECULE: POLY(A) RNA POLYMERASE GLD-2;	(ceGLD-2)
2:	5jnb-B	36.2	2.3	300	324	88	MOLECULE: POLY(A) RNA POLYMERASE GLD-2;	(ceGLD-2)
3:	5jnb-A	36.1	2.1	300	329	87	MOLECULE: POLY(A) RNA POLYMERASE GLD-2;	(ceGLD-2)
4:	5jnb-C	34.2	2.0	275	289	89	MOLECULE: POLY(A) RNA POLYMERASE GLD-2;	(ceGLD-2)
5:	5jnb-D	30.2	1.6	250	263	92	MOLECULE: POLY(A) RNA POLYMERASE GLD-2;	(ceGLD-2)
6:	6l3f-A	26.6	3.4	275	322	27	MOLECULE: UTP:RNA URIDYLYLTRANSFERASE 1;	(atURT1)
7:	5a2w-B	26.4	2.7	278	467	25	MOLECULE: MITOCHONDRIAL PROTEIN;	(ggMTPAP)
8:	4fh3-A	26.4	2.9	278	323	27	MOLECULE: POLY(A) RNA POLYMERASE PROTEIN CID1;	(spCid1)
9:	6l8k-A	26.4	3.3	274	321	27	MOLECULE: UTP:RNA URIDYLYLTRANSFERASE 1;	(atURT1)
10:	4fhv-A	26.3	3.0	278	323	27	MOLECULE: POLY(A) RNA POLYMERASE PROTEIN CID1;	(spCid1)

* The list of neighbours is sorted by Z-score.

Figure S9, Results of Dali search for rnGLD-2, mmGLD-2, and ceGLD-2

Structural homologs of rnGLD-2, mmGLD-2, and ceGLD-2, excerpted from the output of search using Dali Server. 10 candidates with highest Z-score for each search is shown. atURT1, *Arabidopsis thaliana* UTP:RNA uridylyltransferase 1.

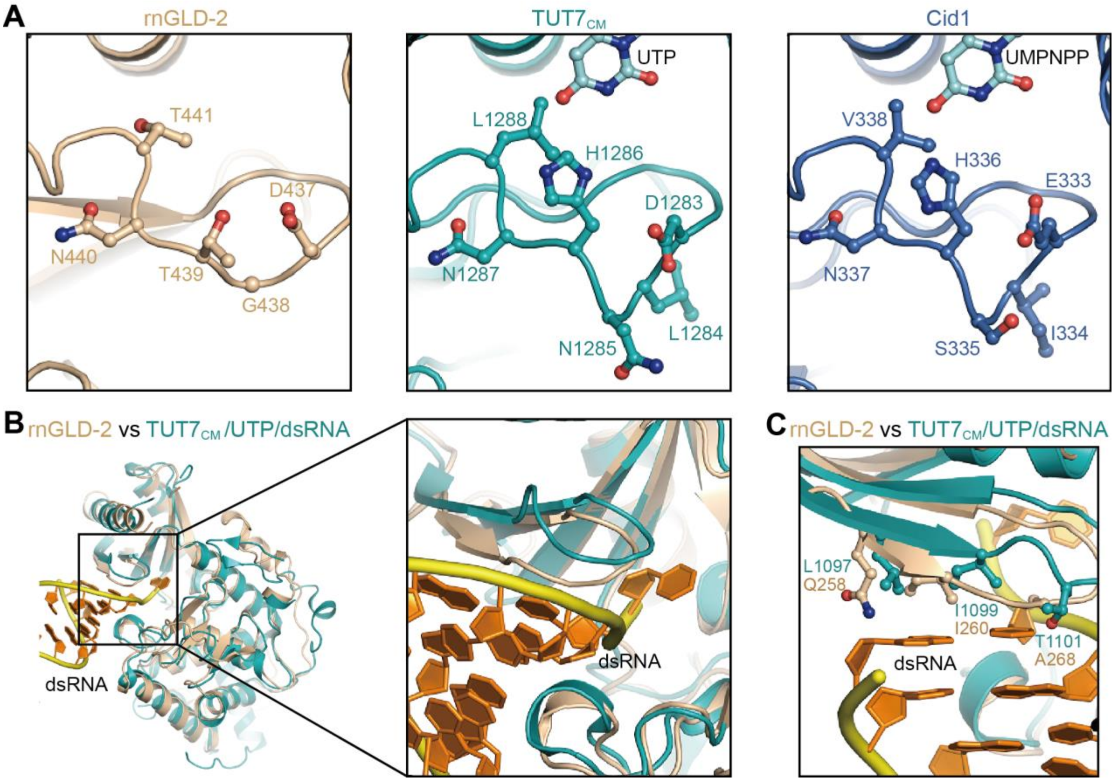

Figure S10, rnGLD-2 structurally resembles TUT7$_{CM}$

(A) Structural comparison of rnGLD-2, TUT7$_{CM}$ (PDB code 5w0o) and Cid1 (PDB code 4nkt) at the NRM region.

(B) Structural comparison between rnGLD-2 and TUT7$_{CM}$/UTP/dsRNA showing the similar folding at the substrate binding site. The substrate dsRNA is partly shown.

(C) Structural comparison between rnGLD-2 and TUT7$_{CM}$/UTP/dsRNA at the hydrophobic platform of the latter that is crucial in positioning the first base pair of the substrate RNA duplex.

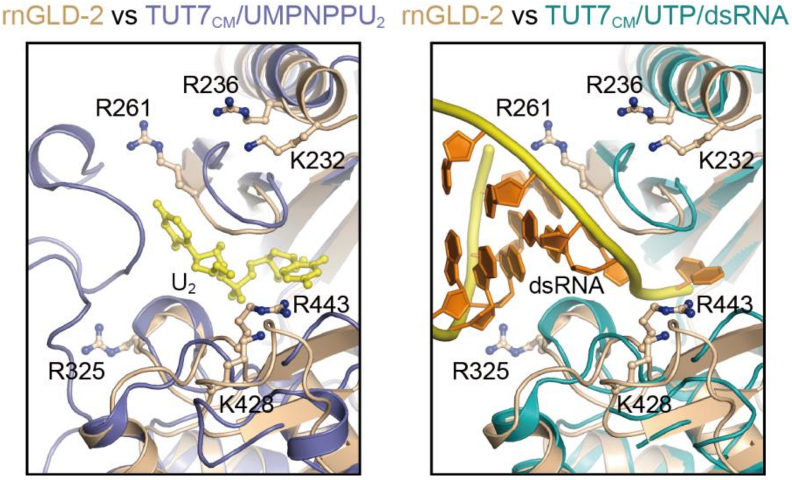

Figure S11, Positively charged surface residues of rnGLD-2 potentially involved in substrate binding

Positively charged surface residues of rnGLD-2 that may be involved in the binding with RNA substrates. rnGLD-2 is superimposed individually with UMPPNP/U_2-bound $TUT7_{CM}$ (left, PDB code 5w0n) and UTP/dsRNA-bound $TUT7_{CM}$ (right, PDB code 5w0o). Residues of rnGLD-2 that may be involved in the interaction with the RNA substrate are shown as ball-and-stick models.

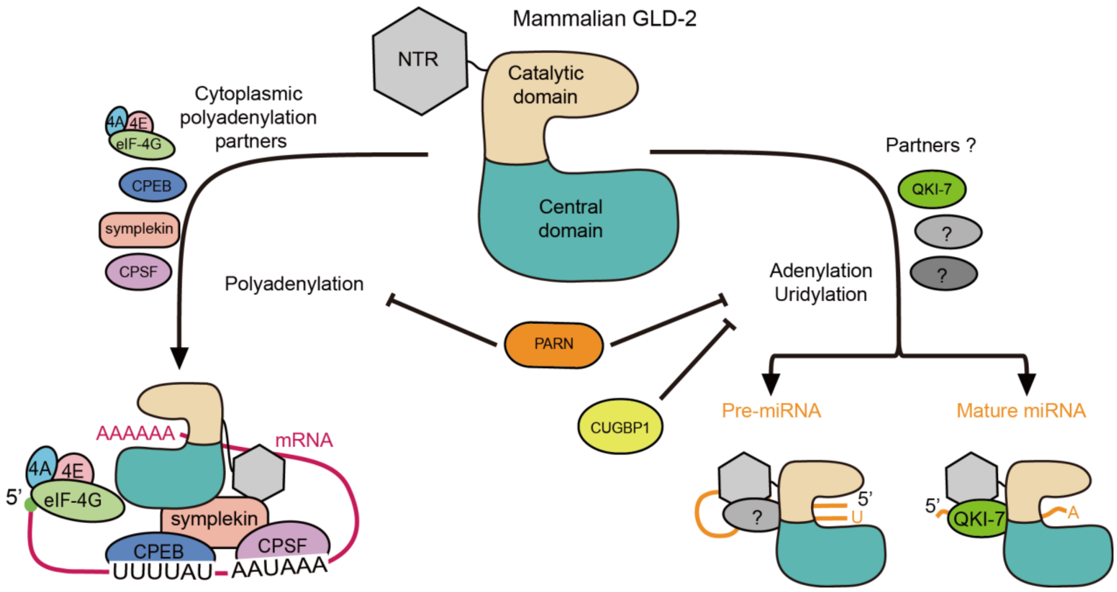

Figure S12, Summary of the cellular function of mammalian GLD-2

Mammalian GLD-2, as an intrinsically potent NTase, is involved in cytoplasmic polyadenylation of mRNAs and adenylation/uridylation of certain mature miRNAs and pre-miRNAs. For mRNA polyadenylation, GLD-2 forms a so-called cytoplasmic polyadenylation complex with other partners including CPEB, CPSF, symplekin, and carry out polyadenylation with the presence of the eukaryotic initiation factor 4A/4E/4G (eIF4A/4E/4G) complex. QKI-7 recruits GLD-2 to miR-122 for mono/oligo-adenylation. It is not clear whether there are other partners involved in the adenylation/uridylation function of mammalian GLD-2 on miRNAs. The deadenylase PARN is known to inhibit the activity of the cytoplasmic polyadenylation complex during mRNA polyadenylation. PARN is also involved in the inhibition of GLD-2-mediated miRNA 3'-end processing together with CUGBP1. NTR denotes the N-terminal region (residues 1–146, see Figure 1) of rnGLD-2.
